# Effects of different hydrological conditions on the taxonomic structure and functional traits of mollusk communities in a large floodplain wetland

**DOI:** 10.1002/ece3.11466

**Published:** 2024-05-26

**Authors:** Yao Zhang, Thibault Datry, Qingji Zhang, Xiaolong Wang, Xianling Xiang, Zhijun Gong, Yongjiu Cai

**Affiliations:** ^1^ Key Laboratory of Lake and Watershed Science for Water Security, Nanjing Institute of Geography and Limnology Chinese Academy of Sciences Nanjing China; ^2^ School of Ecology and Environment Anhui Normal University Wuhu China; ^3^ Collaborative Innovation Center of Recovery and Reconstruction of Degraded Ecosystem in Wanjiang Basin Co‐Founded by Anhui Province and Ministry of Education Wuhu China; ^4^ Poyang Lake Wetland Research Station, Nanjing Institute of Geography and Limnology, Chinese Academy of Sciences Jiujiang China; ^5^ INRAE, UR RiverLy, Centre de Lyon‐Villeurbanne Villeurbanne Cedex France; ^6^ School of Geography and Ocean Science Nanjing University Nanjing China

**Keywords:** flash drought, floodplain wetland, functional traits, hydrological conditions, mollusks, taxonomic structure

## Abstract

Floodplain wetlands are critical to the conservation of aquatic biodiversity and the ecological integrity of river networks. However, increasing drought severity and frequency caused by climate change can reduce floodplain wetlands' resistance and recovery capacities. Mollusks, which are common inhabitants of floodplain wetlands, are among the most vulnerable species to drought. However, the response of mollusk communities to drought has received little attention. Here, we investigated how the structure and functional traits of mollusk communities changed in response to varying hydrological conditions, including a flash drought (FD) in the Poyang Lake floodplain wetland. Our findings showed that FD strongly reduced mollusk abundance and biomass, decreased both α‐ and β‐diversity, and resulted in the extinction of bivalve taxa. A sudden shift in community trait structure was discovered due to the extinction of many species. These traits, which include deposit feeding, crawling, scraping, aerial respiration, and dormancy, help mollusks survive in FD and tolerate completely dry out of their Changhuchi habitat. Finally, we discovered that dissolved oxygen was an important controlling variable for mollusk communities during drought. Our findings provide a scientific basis for the management and conservation of floodplain wetland biodiversity in the context of increasing drought frequency and intensity.

## Graphical abstract

Flash drought (FD) strongly reduced mollusk taxa diversity and led to a sudden shift in the trait structure. Normal drought is a moderate disturbance in floodplain wetland of Poyang Lake. Deposit feeding, aerial respiration and dormancy help mollusks survive in FD.

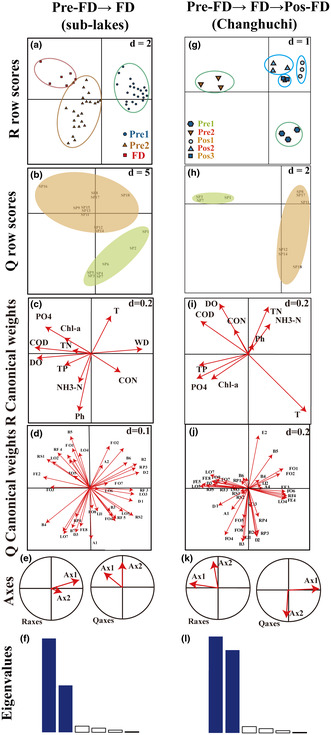

## 1 INTRODUCTION

Floodplain wetlands are one of the most variable landscape features on earth, with high productivity and biodiversity, and they play a crucial role in linking rivers, lakes, and land (Chaparro et al., 2018; Knox et al., 2022). Furthermore, several anthropogenic pressures, like reclamation of land and river regulations, have interfered with the natural flow of water between rivers and floodplains in direct or indirect ways (Dong et al., 2021; Zhang, Pan, et al., 2020). Thus, these activities have made them one of the most vulnerable ecosystems in the world.

Due to human activity and climate change, extreme hydrological events like droughts are occurring more frequently and intensely. This is linked to an increase in the frequency of flash drought (FD), which is caused by a combination of extreme precipitation deficits and abnormally high temperatures (Walker & Van Loon, 2023). FD is characterized by their suddenness and intensity, which typically results in a rapid decrease in local soil moisture and the development of extreme hydrological droughts within only a few weeks. They also cause surface water bodies, like lakes, to dry up quickly and accelerate changes in the hydrological processes of floodplain wetland more quickly (Trenberth et al., 2014; Yuan et al., 2023). Moreover, it substantially decreases vital structural and functional elements of aquatic ecosystems, including nutrient cycling and plant productivity (Chapin & Díaz, 2020; Palmer & Ruhi, 2019).

The features and ecological functioning of floodplain wetlands, including material cycling and energy movement, are influenced by a variety of biotic communities, with macrozoobenthos playing a prominent role (Meena et al., 2019; Nieto et al., 2017). Macrozoobenthos, a group of aquatic animals that spend all or most of their lives on the bottom of a body of water, are also suitable for biological monitoring because they are the first creatures to react to changes in physicochemical factors and available habitats (Koudenoukpo et al., 2021). However, droughts minimize or eliminate benthic habitats during the emergence period, exposing them to a variety of challenges such as dehydration, hypoxia, and starvation (Bond et al., 2008; Humphries & Baldwin, 2003). This has an impact on the growth, development, and reproduction of macrozoobenthos, which alters their life history and causes notable shifts in the dominant species, abundance, and species composition (Poff & Zimmerman, 2010). Extended drought periods may also result in decreased macrozoobenthos resilience and resistance, which frequently takes the form of a simplification of community structure and may cause the loss of ecological functions (Aspin et al., 2019).

In temporary wetlands, community recovery during rewetting normally requires only a few weeks (Datry et al., 2013; Vander Vorste, Corti, et al., 2015). This recovery may be explained in part by the production of drought‐resistant traits like eggs, cysts, and dormant larvae (Datry et al., 2016). For instance, chironomids (Diptera: Chironomidae), which are dominant taxa in many lakes and streams, can survive dry conditions for several months (Larned et al., 2007). However, for long‐lived and less mobile mussel, increasing drought has led to a 60% decline in populations in the Kiamichi River, Oklahoma, USA, over 20 years (Vaughn et al., 2015). Gastropods are one of the most typical and common taxa in wetland macrozoobenthos (Strong et al., 2008) and are particularly sensitive to wetland hydrological processes, especially drought stress, as they need to complete important stages of their life history in water (Wu et al., 2017). Mollusk communities that are periodically subject to drought, such as those in the Mediterranean or arid‐land areas, are highly resistant to desiccation thanks to evolutionary adaptations (Boersma et al., 2013). However, due to the suddenness and intensity of the drought, the resistance of the molluscan community structure from other areas may be significantly reduced or even irreversibly transformed.

The predictions of community resilience to escalating drought are complicated since the underlying mechanisms are regulated by both species tolerance and resistance traits, both of which can be extremely diverse (Bonhomme et al., 2020). Smaller, shorter‐lived, faster‐growing, more generalist taxa close to the bottom of the food web can withstand and recover rapidly from conditions of drought (Aspin et al., 2019; Chessman, 2014). These taxa will most likely be able to access shelter and recolonize rewetted water bodies due to their rapid rate of reproduction and aerial dispersion (Vander Vorste, Malard, & Datry, 2015; Verdonschot et al., 2014). Drought commonly has a negative impact on big, rare specialists near the top of the food chain (Nelson et al., 2021). Most studies, nonetheless, focus on the immediate impact of drought on biodiversity using a standard ‘before‐after’ approach, neglecting recovery tracks that can take time (Bonhomme et al., 2020; Trzcinski et al., 2016).

Α‐diversity, while commonly used to investigate the impact of disturbance on communities, is insufficient for clarifying the underlying mechanisms driving community reactions (Aspin et al., 2019; Ruhi et al., 2017). However, β‐diversity is increasingly being used to comprehend the connection between regional and local patterns of biodiversity and their underlying procedures and is accomplished through analyzing the taxonomic composition of different sites over periods (Aspin et al., 2019; Socolar et al., 2016). Furthermore, a functional approach based on biological trait analyses can supplement taxonomic evaluations by measuring how community‐provided ecosystem functions react to drying events (Gross et al., 2017). For example, Boersma et al. (2013) investigated the relationships between severe pond drying and taxonomic and functional trait diversity in freshwater ecosystem (Boersma et al., 2013).

Mollusks are present in many floodplain wetland ecosystems around the world and are known for their role in water filtration and nutrient cycling (Lopes‐Lima et al., 2018; Vaughn, 2018). In addition, they are also known as good models for floodplain wetland connectivity control due to their diversity in taxonomy, ecological niches, and dispersal capacity (Guan et al., 2017; Hoverman et al., 2011). A large number of studies have been conducted on the ecological effects of wetland hydrological processes, but most of them focus on wetland landscapes, wetland plants, and migratory birds (Jun et al., 2018; Zhang, An, & Leng, 2020), while studies on long‐term water‐demanding mollusks are sparse (Poznańska et al., 2015). Thus, in this study, we explored how the structure and functional traits of mollusk communities varied with contrasted hydrological conditions, including an FD. We expected to see a strong response of mollusk communities to drought, particularly concerning α‐ and β‐diversity. We discussed the implications for the conservation of biodiversity in the floodplain wetland of Poyang Lake.

## 2 MATERIALS AND METHODS

### 2.1 Study area

**FIGURE 1:**
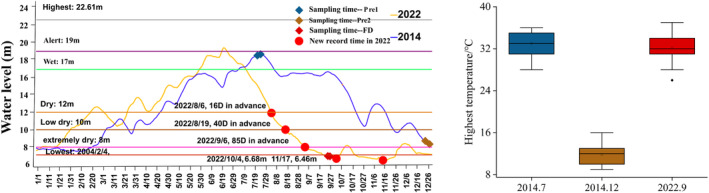
Water level and daily maximum temperature for the sampling month at Xingzi Station, Poyang Lake. Pre1: wet season, Pre2: normal drought, FD: flash drought.

**FIGURE 2:**
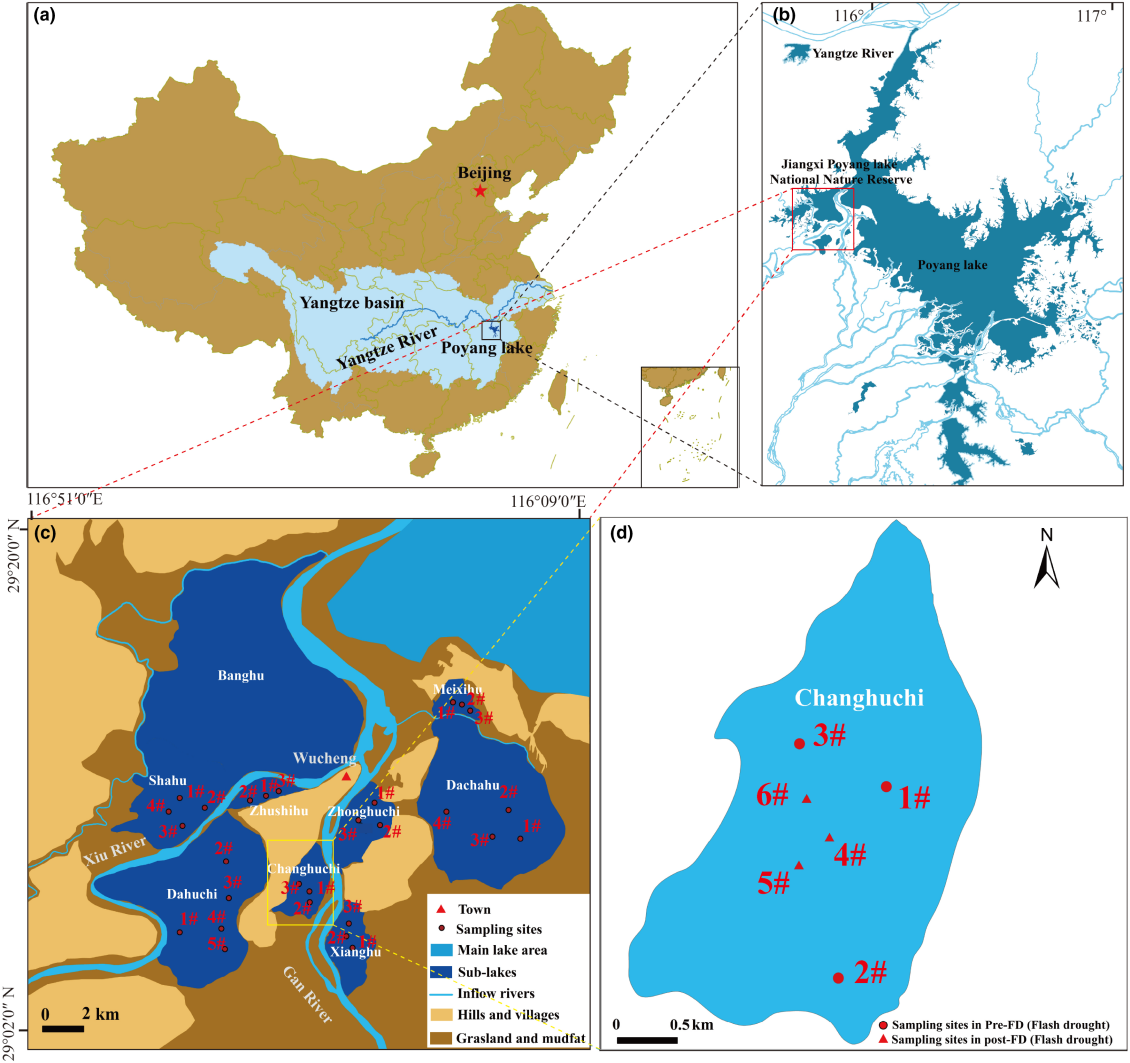
Location of sub‐lakes and the mollusk sampling sites (Table S1). (a) Location of the Poyang Lake. (b) Location of Jiangxi Poyang Lake National Nature Reserve. (c) Location of sub‐lakes and mollusk sampling sites. (d) Mollusk sampling sites in Changhuchi.

Lake Poyang (28°24′–29°46′ N, 115°49′–116°46′ E) lies in the Yangtze River Basin's middle‐lower reaches (Figure 2a). The water level of Xingzi station may even surpass 22.0 m, with a matching lake area of almost 4000 km^2^. In a few typical years, the lake level drops below 8.0 m, and the lake area minimizes to smaller than 1000 km^2^ (Li et al., 2023). Its area has a subtropical monsoon climate with annual precipitation averaging 1400–1900 mm and temperatures averaging 16.7–17.7°C (Mu et al., 2020). Jiangxi Poyang Lake National Nature Reserve has nine sub‐lakes and is located northwest of Lake Poyang (Figure 2b). The conservation area encompasses 224 km^2^ and makes up approximately 5% of the total area of Poyang Lake. Sub‐lakes are seasonal lakes in the lake basin of Poyang Lake. In dry season, the sub‐lakes in the basin are exposed to the shoal, and they are linked with the main lake of Poyang Lake during the wet season and slowly separated from the main lake with a declining water level over the dry season (Feng et al., 2012). The drought that occurred at Poyang Lake in 2022 was an extreme meteorological and hydrological event in the background of global warming; after late June, the Yangtze River basin was under the continuous control of subtropical high pressure, with low precipitation, high temperature and high evaporation, and it took only 7 weeks to drop from the wet water level (17 m) to the extreme dry water level (8 m), so this drought was also a typical flash drought (FD) event (Lyu et al., 2023; Walker & Van Loon, 2023) (Figure 1).

### 2.2 Sampling sites

We sampled three times in different hydrology conditions. According to Figure 1, in July 2014, the period is referred to as wet season with a high water level (>17 m), denoted by Pre1. In December 2014, the water level was at a low water level (8 m) in a normal drought season, the average temperature for the sampling month was 12 °C, and it took about 5 months to go from high water level to a low water level. So, the period is called dry season (normal drought) and is denoted by Pre2. In September 2022, the lowest water level broke the 71‐year record. Additionally, it only took about 8 weeks for the water level (17 m) to fall to the extremely dry water level (8 m), so we think this drought belongs to the flash drought event, which is denoted as FD. Normal drought in Pre2 at Poyang Lake occurs every year, but FD is the most severe drought event in the past 71 years.

To explore mollusk dynamics from pre‐FD to FD, we collected mollusks from 8 sub‐lakes in Poyang Lake National Nature Reserve in the three sampling periods. In July 2014, we collected 2–5 sampling sites according to the water area and habitat condition of each sub‐lake, and the water surface area of sub‐lakes was larger in Pre1 with higher water levels, and we collected mollusks in 3–5 sampling site for each sub‐lake, and totally 27 sampling sites were sampled. In December 2014, a total of 24 sample sites were collected due to decreased water level and water surface area. In September 2022, the water surface area was very small owning to FD, and only 1 sample site in the center part of each lake was collected, among which Xianghu and Changhuchi were completely dried up and no mollusks were collected, and a total of 6 sample sites was collected.

To explore the community dynamics of mollusks after FD, we selected one of the sub‐lakes (Changhuchi) as the specific area. Changhuchi dried up completely on 6th September 2022 and remained for about 20 days, Changhuchi started rewetting on 26th September 2023, and then the water depth was maintained at about 30 cm. Therefore, we conducted 3 follow‐up monitoring within 5 months of rewetting in Changhuchi. In addition, we denote rewetting 1 month as Pos1, rewetting 2 months as Pos2, and rewetting 5 months as Pos3. Due to the small surface area of Changhuchi Lake, the original sampling sites were moved toward the center of the lake, and three new sampling sites were identified (Figure 2).

### 2.3 Sampling methods and measurement of physicochemical parameters

Three repeated mollusk samples were obtained at each sampling site using a D‐type net and modified Petersen grab (area of 1/16 m^2^). The mollusks were carefully selected from the sediment and placed in a white porcelain dish before being preserved in 10% ethanol. The mollusks were numbered, weighed, and converted to ash‐free dry weight utilizing an electronic balance. Before the statistical analysis, the three duplicate samples of each site from a modified Petersen grab or D‐shaped net were combined and transformed to abundance each square meter.

A YSI 6600 V2 Multi‐Parameter Water Quality Sonde was used to measure temperature (TEMP), dissolved oxygen (DO), pH, and conductivity (CON). Ammonium (NH3‐N), Total nitrogen (TN), orthophosphate (PO4‐P), total phosphorus (TP), chlorophyll a (Chl‐a), and permanganate index (COD_Mn_) were measured in the laboratory according to APHA standards (Rice et al., 2012).

### 2.4 Data analysis

To describe the taxonomic diversity of mollusk communities, we used total mollusk abundance and species richness. Mollusk species richness was calculated using the Shannon‐Wiener index and the Simpson index (Magurran, 2021). For each hydrological period, the presence‐absence Jaccard index was calculated and separated into two additional components, replacement and richness difference (Legendre & Condit, 2019). Non‐metric multidimensional scaling (NMDS) was used for analyzing molluscan species composition, and significant variations in molluscan species composition between hydrological periods were investigated via permutational multivariate analysis of variance (PERMANOVA) (Habagil, 2019).

We calculated four indices weighted by species abundances, namely functional richness (FRic) (Villéger et al., 2008), functional evenness index (FEve) (Mason et al., 2005), functional dispersion (FDis) (Prada‐Salcedo et al., 2021), and functional divergence index (FDiv) (Díaz & Cabido, 2001). Then we estimated Simpson's (D) and Rao's (Q) diversity and calculated Functional Redundancy (FRed) (Ricotta et al., 2016). Kruskal–Wallis and Dunn's post‐hoc tests were used to compare abundance, biomass, α‐diversity, β‐diversity, and functional diversity among different hydrological periods in sub‐lakes. Because mollusks were not collected at some sites in the Changhuchi during periods of FD and Pos‐FD, we used the average of abundance at the three sites in the Changhuchi to calculate α‐diversity, β‐diversity, and functional diversity. Therefore, Kruskal–Wallis and Dunn's post‐hoc tests can only be used to compare abundance and biomass during different hydrological periods in Changhuchi. The abovementioned analysis was performed for R software.

We used RLQ and fourth‐corner approaches to investigate relationships between mollusk functional traits and driver factors in different hydrological conditions and rewetting durations. RLQ analysis and the fourth‐corner method are currently the most integrated approaches for analyzing the connections between functional traits and environmental factors (Dray et al., 2014; Kleyer et al., 2012). Both approaches examine data from three tables at the same time: an environmental parameters table a species abundance table, and a functional traits table (Dolédec et al., 1996; Dray et al., 2014). Before performing the RLQ analysis, the three tables were examined separately. The species abundance table was subjected to correspondence analysis (CA), and the functional traits table was subjected to principal component analysis (PCA) (Dray et al., 2014). We used Hill‐Smith analysis since the environmental variables table included both category and quantitative variables. A global Monte‐Carlo test with 49,999 random permutations of model 2 and model 4 was used to assess the overall significance of this connection. To evaluate the relationships between species traits and environmental variables, the fourth‐corner analysis was used. However, Dray et al. (2014) identified several disadvantages only associated with RLQ or the fourth‐corner analysis (Dray et al., 2014). Thus, combining RLQ and the fourth‐corner could conquer these disadvantages. The R software package ade4 was used to perform the RLQ and fourth‐corner analyses.

## 3 RESULTS

### 3.1 Taxonomic diversity

**FIGURE 3:**
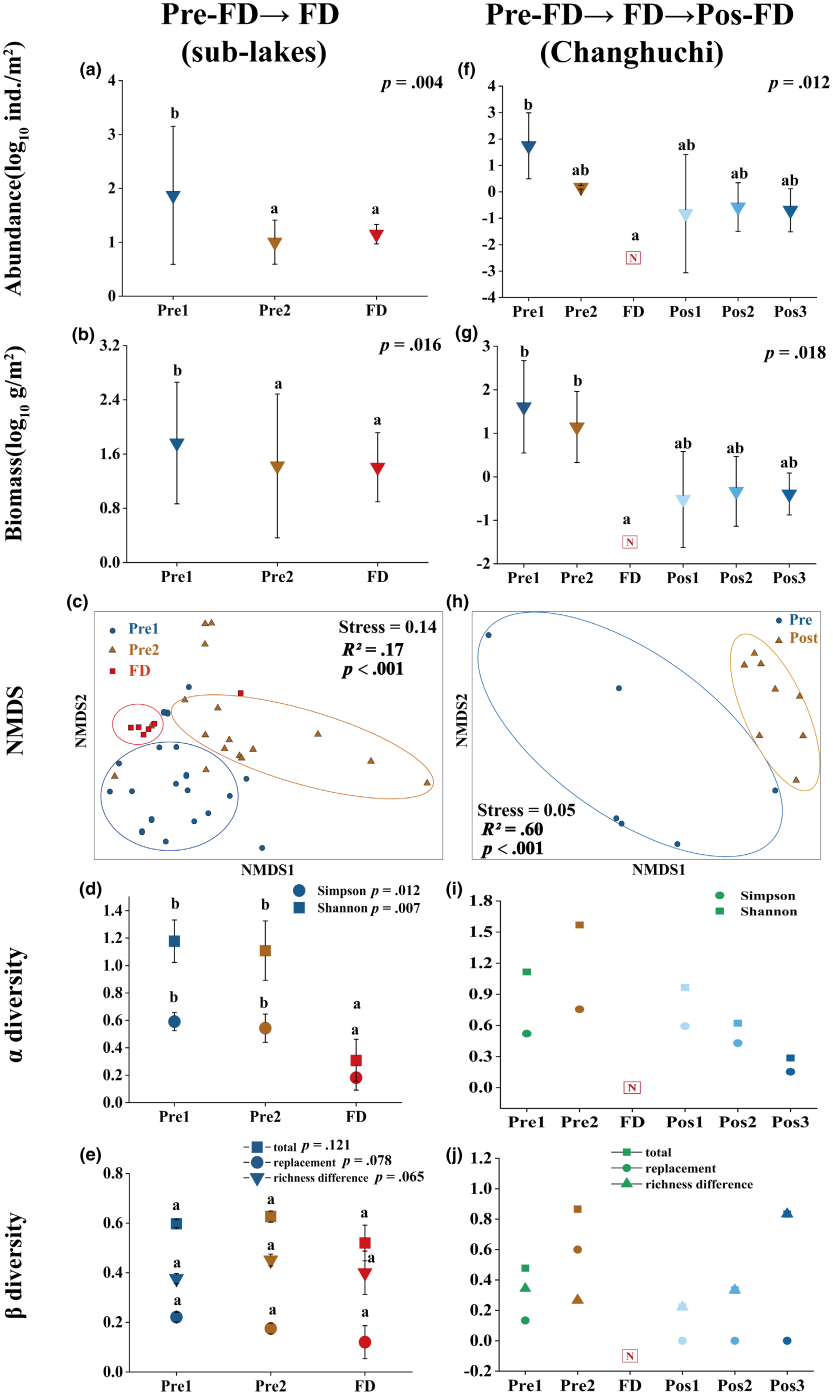
Temporal variation of mollusks taxonomic diversity in sub‐lakes and Changhuchi. Pre‐FD → FD: from before flash drought to flash drought; Pre‐ FD → FD → Pos‐FD: from before flash drought to flash drought to after flash drought; Pre1: wet season, Pre2: normal drought, FD: flash drought, Pos1: rewetting 1 month, Pos2: rewetting 2 months, Pos3: rewetting 5 months. Blow same. p (a, b, d–g): results from the Kruskal–Wallis test, with *p* < .05 representing a significant difference between different periods. Different letter superscripts: from results of the Dunn's post‐hoc tests, indicating significant differences between different periods (*p* < .05). p (c, h): result of PERMANOVA, *p* < .05 represents significant difference between different groups. The letter N in (f) and (g): no surviving individuals; The letter N in (i) and (j): because of no surviving individuals, α‐diversity and β‐diversity could not be calculated.

There were significant variations in the biomass (*p* = .016) and abundance (*p* = .004) of mollusks from pre‐FD to FD (sub‐lakes), both with *p* < .05. The abundance of mollusks in Pre1 was the highest (82.04 ind./m^2^), followed by FD (10.63 ind./m^2^); the abundance of mollusks in Pre2 was the lowest (9.93 ind./m2) (Figure 3a). Mollusk biomass varied greatly, with the highest value in Pre1 (58.85 g/m^2^), the second‐highest in Pre2 (26.10 g/m^2^), and the lowest in the FD (19.06 g/m^2^) (Figure 3b). PERMANOVA revealed significant differences in mollusk assemblage structure among different periods (Figure 3c). The abundance of *Bellamya aeruginosa* was greatest in different periods. In Pre1, the abundance of *B aeruginosa* (225.16 ind./m^2^), *Parafossarulus striatulus* (85.24 ind./m^2^), and *Alocinma longicornis* (170.49 ind./m^2^) were greater than other species, and the biomass of *B. aeruginosa* (255.68 g/m^2^), *Corbicula fluminea* (130.17 g/m^2^), and *A. longicornis* (20.70 g/m^2^) was greater than other species. In Pre2, the abundance of *B. aeruginosa* (32.81 ind./m^2^), *P. striatulus* (10.82 ind./m^2^), *Parafossarulus sinensis* (15.19 ind./m^2^), and *A. longicornis* (12.44 ind./m^2^) was greater than other species. The biomass of *Anodonta woodiana elliptica* was highest with a value of 99.42 g/m^2^, followed by *B. aeruginosa* (70.78 g/m^2^). In FD, Bivalvia were not found, and the abundance and biomass of *B. aeruginosa* (57 ind./m^2^, 120.84 g/m^2^), *Parafossarulus eximius* (14.00 ind./m^2^, 11.26 g/m^2^), and *Radix swinhoei* (10.00 ind./m^2^, 1.30 g/m^2^) was greater than other species (Table S2). With the increase in drought abundance, Simpson, Shannon, and replacement all showed a decreasing trend, and Simpson and Shannon decreased significantly in the FD period (*p* < .05). Spatial taxonomic β‐diversity and richness differences were greatest in Pre2, followed by Pre1, and the β‐diversity of FD period was lowest (Figure 3d,e). Spatial taxonomic richness difference contributed more than a replacement to taxonomic β‐diversity among different hydrological periods.

In Changhuchi (from Pre‐FD to Pos‐FD), the abundance and biomass in Pre1 and Pre2 were greater than those in other times, and no survival individuals were found in FD. Few survival individuals were found after Pos1, and then the abundance and biomass showed fluctuating trends (Figure 3f; Figure 3g). Furthermore, a PERMANOVA study of the assemblage structure of mollusks dependent on abundance showed significant variations between pre‐FD and post‐FD (*p* < .001) (Figure 3h). We also found significant differences in the abundance (*p* = .012) and biomass (*p* = .018) among different times in Changhuchi (*p* < .05). In Changhuchi, the abundance of *B. aeruginosa* (37.33 ind./m^2^) was greatest in Pre1, followed by *B. moellendorff* (5.33 ind./m^2^) and *P. striatulus* (5.33 ind./m^2^), and the abundance of *C. fluminea* (1.06 ind./m^2^) was lowest; in Pre2, the abundance of *A. longicornis* (0.53 ind./m^2^) and *U. douglasiae* (0.43 ind./m^2^) was greater than other species, and the abundance of *B. aeruginosa* (0.11 ind./m^2^) and *P. eximius* (0.11 ind./m^2^) was lowest. No survival individuals were found in FD. *C. chinensis* (0.02 ind./m^2^), *B. aeruginosa* (0.07 ind./m^2^), and *P. eximius* (0.07 ind./m^2^) were found after Pos1 but in slow abundance. Only *B. aeruginosa* and *P. eximius* were found after Pos2 and Pos3 and at slow abundance. The biomass of *B. aeruginosa* was highest in Pre1 with a value of 34.57 g/m^2^, followed by *C. fluminea* with a value of 2.75 g/m^2^, and the biomass of *C. fluminea* (0.34 g/m^2^) was lowest; The biomass of *Unio douglasiae* was highest in Pre2 with a value of 8.29 g/m^2^, followed by *Anodonta woodia. pacifica* with a value of 5.14 g/m^2^, and the biomass of *C. fluminea* (0.01 g/m^2^) were the lowest. The biomass of *B. aeruginosa* was highest after rewetting, and *C. chinensis* was just found after Pos1 (Table S3). Changhuchi, Simpson, and Shannon all showed an increasing trend from the Pre1 to the Pre2 and decreased with increasing rewetting time. Spatial taxonomic β‐diversity and replacement showed an increasing trend from Pre1 to the Pre2 and spatial taxonomic β‐diversity and richness difference showed an increasing trend with increasing rewetting time. And only spatial taxonomic richness difference contributed to taxonomic β‐diversity. Since no surviving individuals were found in FD, diversity could not be calculated (Figure 3i,j).

### 3.2 Functional diversity

**FIGURE 4:**
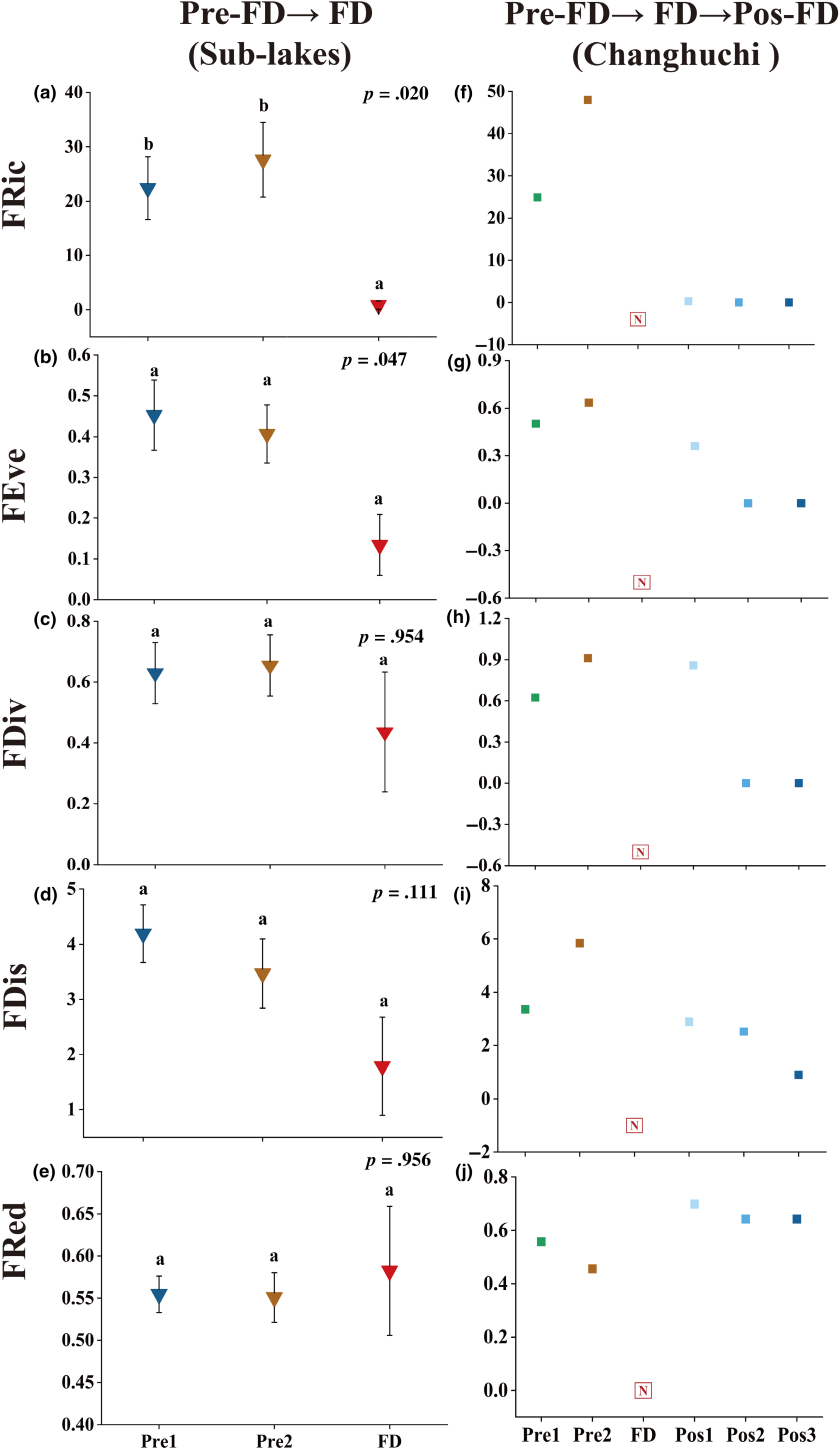
Function diversity of mollusks in sub‐lakes and Changhuchi. *p*: results from the Kruskal–Wallis test, with *p* < .05 representing a significant difference between groups; Different letter superscripts: from results of the Dunn's post‐hoc tests, indicating significant differences between different periods (*p* < .05); The letter N: because of no surviving individual functional diversity could not be calculated.

In sub‐lakes (From FD to Pos‐FD), FRic was significantly higher in Pre1 and Pre2 compared to FD (*p* < .05). FRic and FDiv reach a maximum in the Pre2, followed by a decreasing trend and a minimum in FD. The increase in drought intensity dropped the FEve and FDis, which reached their minimum values at FD. From FD to Pos‐FD, FRed shows an increasing and then decreasing trend and reaches a maximum at FD. In Changhuchi (From Pre‐FD to Pos‐FD), FRic, FEve, FDis, and FDiv all reach a maximum in Pre2, but function diversity could not be calculated due to no survival individuals in FD. Function diversity began to recover after Pos1, but values of FRic and FDis in the FD are less than 60% of those in Pre2. With increasing duration of rewetting, diversity tends to decrease and FRic, FEve, and FDiv reach a value of 0 at Pos2. The FRed of Pos‐FD were all greater than those of Pos‐FD (Figure 4).

### 3.3 RLQ and fourth‐corner analysis

**FIGURE 5:**
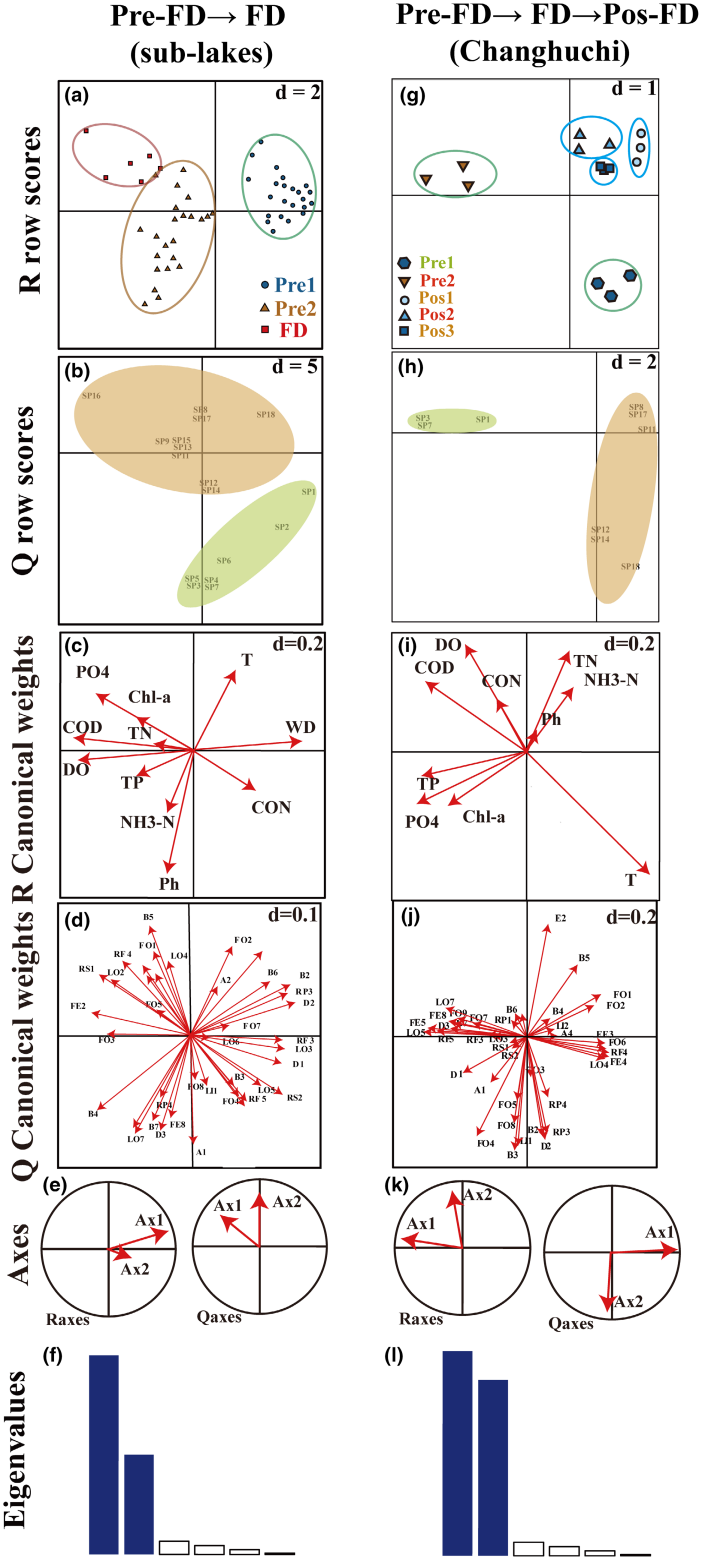
The results of the first two axes of the RLQ analyses in sub‐lakes and Changhuchi. Sample site variation (a; g), species variation (b; h), environmental variation (c; i), and trait modalities variation (d; j). Relationships between R axes and RLQ axes (e; k); eigenvalues, with the first two axes highlighted in blue (f; l). Table S4 contains abbreviations; Table S5 contains species abbreviations; and Tables S6 and S7 contain environmental factor abbreviations.

From Pre‐FD to FD (sub‐lakes), the global testing process only found significant for model 2 (model 2: *p* = .005; model 4: *p* = .289). The first and second RLQ axes explained 55.82% and 29.25% of the total variation, respectively. Figure 5e,f show the eigenvalues and relationships of the R, Q, and RLQ axes, respectively. The RLQ analysis revealed sites with increasing DO and COD gradients to drought intensity, with the greatest levels at FD (Figure 5a,c). The corresponding species (e.g., *R swinhoei*) were deposit feeders (FE2) and tegument respirers (RS1; Figure 5b,d). The right part of the first RLQ axis shows trait modalities associated with WD (species associated with reproduction via isolating and cementing eggs (RP3), aquatic passive (D1), and full water swimmer (LO3)). These trait modalities closely matched those of *C. fluminea*, *Limnoperna fortune*, and *Bithynia fuchsiana Moellendorff*. The trait modalities respiration through tegument (RS1) and deposit feeder (FE2) were associated with DO and COD in the negative region of the first RLQ axis. The main respective species were *R. swinhoei* (Figure 5b,d).

The global test process was significant from pre‐FD to Pos‐FD (Changhuchi) (model 2: *p* = .0005; model 4: *p* = .0024). The first RLQ axis explained 89.14% of the total variation, while the second RLQ axis accounted for only 7.49%. Figure 5k,l also shows the eigenvalues and relationships of the R, Q, and RLQ axes. The RLQ analysis identified sites with increasing DO gradients from Pre‐ to Post‐FD, with the highest levels at Pos1, and the corresponding species (e.g., *B. aeruginosa*, *C. chinensis*) feeding on fine sediment, microorganisms (FO1), and detritus (1 mm) (FO2) (Figure 5g,i). The negative section of the first RLQ axis indicates trait modalities (burrower (epibenthic) (LO5), filter feeder (FE5), and none resistance form (RF5)) associated with COD, TP, and PO4. These trait modalities were most closely related to the traits of the bivalve *C. fluminea*, *A. elliptica*, and *Unio douglasiae*. The right region of the first RLQ axis showed trait modalities (diapause or dormancy (RF4), scraper (FE4), and feeding on dead animal (>1 mm) (FO6)) associated with T, (crawler) LO4, and (shredder) FE3 associated with T. The main respective species were *B. aeruginosa*, *P. eximius*, and *C. chinensis* among gastropoda. The trait modalities (deposit feeder (FE2)) associated with DO were shown in the positive region of the second RLQ axis (Figure 5h,j). The main respective species were *B. aeruginosa*.

**FIGURE 6:**
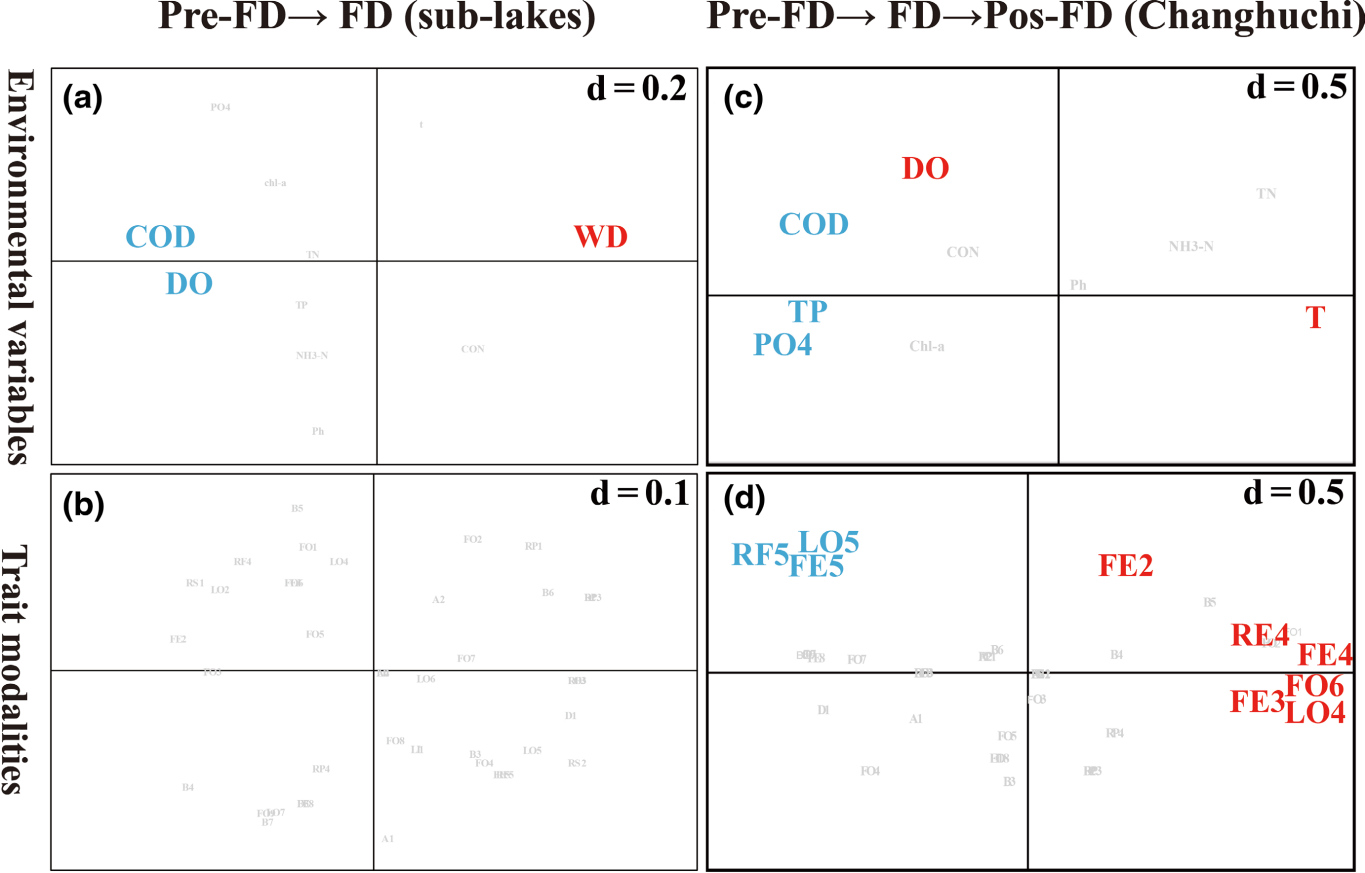
Significant relationships between trait modalities (a; c) and environmental variables (b; d). COD: Chemical Oxygen Demand; DO: Dissolved Oxygen; WD: Water Depth; TP: Total Phosphorus; PO4: Phosphate; T: Water Temperature; RF5: none (Resistance form); LO4: crawler (Locomotion and substrate relation); LO5: burrower(epibenthic) (Locomotion and substrate relation); FO6: dead animal > 1 mm (Food); FE2: deposit feeder (Feeding habits); FE3: shredder (Feeding habits); FE4: scraper (Feeding habits); FE5: filter feeder (Feeding habits); The red font represents positive correlation, and the blue font represents negative correlation.

We then combined RLQ and fourth‐corner analysis to investigate for significant bivariate relationships between RLQ axes and trait modalities/environmental parameters. The AxcQ1 had a significant positive correlation with WD from Pre‐FD to FD (sub‐lakes). COD and DO were significantly negatively related to AxcQ2 (Figure 6a). The AxcQ1 had a significant positive correlation with temperature and was significantly negatively related to COD, TP, and PO4 from Pre‐FD to Pos‐FD (Changhuchi). The AxcQ2 was significantly positively correlated with DO (Figure 6c). Species attached to diapause or dormancy (RF4), scraper (FE4), and feeding on dead animal (>1 mm) (FO6), (crawler) LO4, and (shredder) FE3 were significantly positively associated with the AxcR1 and species related to burrower(epibenthic) (LO5), filter feeder (FE5) and none resistance form (RF5) were significantly negatively associated with the AxcR1. And AxcR2 was significantly positively associated with (deposit feeder) FE2 (Figure 6d).

## 4 DISCUSSION

### 4.1 Changes in the taxonomic structure of mollusk before and after flash drought

Drought reduces mollusk abundance significantly, which is consistent with previous research (Aspin et al., 2019). They did not have enough time to join the water column since the water withdrew quicker than the mollusks could migrate during the dry season (Pre2). The extremely quick pace of recession and severe temperatures, particularly during flash drought (FD), exacerbated molluscan mortality (Figure S1) (Chase, 2007; Trzcinski et al., 2016). Molluscan density and biomasses, however, were comparable during normal drought and FD times, which is likely owing to mollusk concentrations in diminishing habitats (Herbst et al., 2019). Changhuchi may not have shown a rising trend of mollusks after rewetting since autumn and winter are not appropriate for mollusk development and reproduction.

Furthermore, drying caused considerable alterations in the mollusk assemblage structure. Normal drought did not reduce α‐diversity since it attenuates interspecific competition by reducing mollusk densities and instead helps to increase community resistance. In contrast with normal drought, FD causes the disappearance of bivalve taxa. This could be because bivalves are less resistant to and recover from drought than gastropods, and the high temperatures mixed with the once‐in‐a‐century drought led to the loss of bivalve species. Mollusks, in particular, are ectotherms and hence highly vulnerable to high temperatures, and this effect is expected to be accentuated in floodplain wetlands where shallow water levels may allow a more rapid reaction to strong heat occurrences in FD (Epele et al., 2022). The study of the effects of FD (high heat and extreme drought) on the structure of mollusk communities has crucial implications for the impact of climate change on biodiversity (Vander Vorste, Corti, et al., 2015).

We discovered that richness differences in different hydrological periods of sub‐lakes were the main contributors to β‐diversity. This is comparable to Wang et al.'s study on the impact of dams on riverine benthic communities (Wang et al., 2021). Sub‐lakes are interconnected for an extremely brief duration (water level > 17 m), and the environmental conditions of the dry period may have acted as a dispersal barrier, preventing mollusk migration and thus resulting in richness differences contributing more to β‐diversity than replacement (Heino et al., 2015). Although β‐diversity increased with rewetting duration, it was only due to richness difference. This is similar to the decline in inter‐community replacement caused by the dam's division of the intermittent stream, where FD resulted in the complete drying out of the Changhuchi and the formation of crevasses that inhibited mollusk dispersal and thus reduced replacement. In contrast, β‐diversity in Changhuchi was driven by replacement in Pre2, because normal drought did not produce cracks that hinder mollusks from colonizing suitable habitats (Heino et al., 2014).

### 4.2 Changes in the functional diversity of mollusk before and after flash drought

Normal drought increased FRic and FDiv since drought reduced molluscan abundance, allowing mollusks to occupy more ecological space. Mollusks had to reduce interspecific competition through ecological niche differentiation as water area decreased. Mollusks reduced interspecific competition during the wet season (Pre1) primarily by increasing dispersal (Mammola et al., 2021). Both species and functional diversity displayed similar patterns in the Pre1 and Pre2, mostly following the patterns predicted by the intermediate‐disturbance hypothesis (Leigh, 2013). Environmentally harsh systems, such as floodplain wetlands, which have historically endured normal drought, harbor species possessing traits that foster resistance to disturbance, resulting in a high functional diversity (Boersma et al., 2013; Vander Vorste, Corti, et al., 2015). However, the loss of most species in FD caused an abrupt change in the trait structure of communities, resulting in a astringent of functional traits of surviving taxa. Indeed, we found that most species decreased in number during FD, merely the most resistant species kept (*B. aeruginosa*, *P. eximius*, *P. sinensis*, *C. chinensis*). This indicates that the taxonomic structure composition is susceptible to FD and supports the view that niche‐selection sorting, instead of randomness, decides the functional pattern of communities over drought (Chase, 2007). Communities were unable to return to pre‐FD conditions during the rewetting period, indicating a loss of resilience (Bogan & Lytle, 2011). The reason for the loss of resilience could be that FD outweighs the behavioral and physiological responses of species to drought. The rewetting of Changhuchi revealed that only a few species can survive the remaining moisture and endure dehydration in the stage of adulthood. The loss of taxa with these traits leads to decreases in functional diversity. Climate change‐induced increases in drying duration and intensity may reduce the number of nearby perennial shelters and resources for colonists, as well as reduce hydrological interconnectivity (Jaeger et al., 2014). Long‐term monitoring is thus required to evaluate the ongoing alteration of floodplain wetland and the biological implications for community reconstruction after drought events.

High resistance to taxonomic variety is one way for communities to protect themselves from environmental extremes; functional trait redundancy is another. Functional redundancy is a measure of the extent to which taxonomically different species perform similar ecological roles within an ecosystem or share comparable features (Rosenfeld, 2002), and it can supply ecosystems with some protection against the loss of functioning of ecosystems caused by species extinction (Petchey et al., 2007; Philpott et al., 2012). Environmentally harsh systems, like the Poyang Lake floodplain wetland, which has previously experienced drought events, are home to taxa that have traits that boost resistance and resilience to disturbance, resulting in a high degree of functional redundancy (Boersma et al., 2013; Vorste et al., 2016). We anticipated less functional redundancy in FD when taxa did not face such selective pressure. However, in FD, communities demonstrated greater functional redundancy. Assuming that functional redundancy has a positive relationship with ecosystem resilience, FD ought to boost ecosystem resilience (Bruno et al., 2016). This large disparity is most likely due to differences in trait choice, programming, and analytical methods in functional redundancy patterns described by others in aquatic systems (Crabot et al., 2021). However, there are fewer findings for floodplain wetlands. More efforts are needed to describe functional redundancy patterns in floodplain wetlands, as well as conceptual work to explain underlying mechanisms.

### 4.3 RLQ and fourth‐corner analysis

The RLQ analysis indicated that as dry intensity increased, the functional trait structure of the mollusks changed owing to the absence of bivalves in FD. Some of these species shared functional traits, which might give rise to functional redundancy in community. Previous research has found that macrozoobenthos community possessing functional redundancy could be less vulnerable to alterations in aquatic ecosystem functioning caused by the extinction of species (Gamfeldt et al., 2008; Nasi et al., 2018). However, this study discovered that functional over‐redundancy may have negative consequences. Functional over‐redundancy implies functional vulnerability because these comparable attributes are not resistant to FD (Mouillot et al., 2014). Previous research has found a link between body size and resistance to desiccation, because larger creatures have a lower surface area to volume ratio than smaller ones, making them less susceptible to water loss (Bujan et al., 2016). Previous research has also found that big taxa are vulnerable to the FD in floodplain wetland (Ledger et al., 2013). However, we should note that the findings of this study revealed that individuals' body size did not have a more significant correlation with drought. Some species' extinction is caused by their drought resistance and habitat preferences. Individuals with varying body sizes, such as *A. elliptica*, *U. douglasiae*, and *C. fluminea*, were not resistant to FD. For example, different body sizes individuals such as *A. elliptica*, *U. douglasiae*, and *C. fluminea* were not resistant to coping with FD. *P. striatulus* and *A. longicornis* inhabited aquatic plants, and FD destroyed their habitats, leading to their disappearance, whereas gastropods of similar size such as *B. aeruginosa*, *P. eximius*, *C. chinensis*, and *R. swinhoei* were able to survive in FD through characteristics such as dormancy, and thus body size contribute less to molluscan resistance to FD in the floodplain (Miller et al., 2021). The resistance of macrozoobenthos to droughts usually depends on the dispersal by aquatic stages (Chester & Robson, 2011). However, our results do not prove the standpoint, since both bivalves and gastropods have poorer dispersal ability compared to other macrozoobenthos with lower scores, and therefore mollusks' dispersal ability is less useful for escaping from normal drought and FD.

In Pre1 and Pre2, the deposit feeder and filter feeder were observed. However, the deposit feeder was only observed in FD and rewetting. Similarly, Dézerald et al. also found that species feeding on detritus are among the numerically dominant within food webs of ecosystems (Dézerald et al., 2016). The reason for this may be that in sub‐lakes, the high temperatures and very low water levels (20 cm) during the FD period resulted in the death of a large number of aquatic plants, which in turn provided a food source for the deposit feeder. In particular, the completely dry Changhuchi promoted the growth of drought‐tolerant wetland vegetation, but after rewetting, the wetland plants were submerged and died, again providing a food source for deposit feeders. Our findings showed that aerial respiration assisted Gastropodas (e.g., *R. swinhoe*) in surviving FD because they were less affected by low oxygen levels in drying aquatic habitats (Nelson et al., 2021; Vorste et al., 2016). Gastropoda with dormancy ability was discovered during rewetting, which is consistent with the report of Nelson et al., perhaps because lots of cracks also helped Gastropoda have suitable dormant habitats to survive in dried Changhuchi (Figure S2) (Nelson et al., 2021). No individuals with the burrower trait were found to survive in the Changhuchi after rewetting because the FD hardened the Changhuchi substrate and immobilized these individuals in the substrate, and even if these individuals survived the FD, they died during rewatering due to the inability to feed (Figure S3). The general transition toward desiccation‐resistant species was consistent with a transition from full‐water swimmers and burrowers to species that crawl in moist detritus (Dézerald et al., 2015). This is similar to the findings of Dézerald et al. (2015). Because the full water swimmer and burrower were filter‐feeders of microphytes and microzoobenthos, but crawlers are largely detritivores, FD could promote a transition from a microphytes‐mollusks‐based to a detritus‐based food chain. This type of drought‐induced alteration of habitat‐related traits has been found frequently in aquatic communities (Bonada et al., 2007; Dézerald et al., 2015; Diaz & Rosenberg, 2008), giving rise to the notion that habitat use is an excellent indicator for drying resistance (Robson et al., 2011).

Alterations in taxonomic composition have been related to both local and large‐scale environmental factors including dissolved oxygen, total nitrogen, and ammonia. Because dissolved oxygen is closely linked to water quality, our study further demonstrated that it was a controlling factor for mollusk communities (Dolédec et al., 2021). As algal sources within the floodplain wetland gathered on available substrates, the initial availability of nitrogen promoted the survival of scrapers or detritivores (e.g., Gastropods) (Cornut et al., 2012; Ledger & Hildrew, 2005). The RLQ and fourth‐corner analyses revealed that some functional traits reacted to drought. However, conclusions about these connections should be drawn with caution (Gusmao et al., 2016). We found that floodplain wetland characteristics like local climate and hydrology can have a big impact on mollusk resistance and recovery. As future climate scenarios predict an increase in the frequency and intensity of climate droughts in floodplain wetlands, environmental managers must anticipate how species will cope with drought to support climate change adaptation (Cereghino et al., 2020).

## 5 CONCLUSION

The drought in 2022 was the most severe drought event (flash drought (FD)) at Poyang Lake in 71 years, resulting in a complete drought in 2 sub‐lakes in Poyang Lake National Nature Reservation. Thus, we analyzed the taxonomic diversity, functional diversity, and the relationship between functional traits and drought before and after the FD.

Our results revealed that FD strongly reduced mollusk taxonomic diversity and led to the loss of bivalve taxa. Since less connectivity between the sub‐lakes inhibited mollusk dispersal, β‐diversity was mainly contributed by richness difference. There was a sudden shift in the trait structure of communities due to the loss of most species in FD, but FD increased the functional redundancy of the molluscan community. Normal drought may be a moderate disturbance that reduced interspecific competition by reducing mollusk abundance and thus preserving the functional traits of species. In addition, these traits, including deposit feeding, crawling, scraping, aerial respiration, and dormancy, not only help mollusks survive in FD but also tolerate the complete dry out of their habitat in the Changhuchi.

Because suitable hydrological connectivity promotes higher β‐diversity, measures of management should be undertaken to keep water during the withdrawal period to maintain a certain level of connectivity as well as the scope of surface water in sub‐lakes. In addition, monitoring of water levels and early warning devices for abnormal levels should be arranged to avoid the effects of complete drying of sub‐lakes on mollusks. A mechanistic understanding of ecosystem resilience to drought events may help scientists and environmental managers mitigate the impacts of drought by focusing on relevant levels of species and metacommunities (Bonhomme et al., 2020). However, in this study, mollusks did not return to their pre‐FD state during the 5 months after FD, so continuous field monitoring after FD is necessary, and the resilience of mollusks to cope with FD has yet to be investigated.

## AUTHOR CONTRIBUTIONS

**Yao Zhang:** Conceptualization (equal); data curation (equal); formal analysis (equal); investigation (equal); methodology (equal); visualization (equal); writing – original draft (equal); writing – review and editing (equal). **Thibault Datry:** Data curation (equal); formal analysis (equal); investigation (equal); methodology (equal); supervision (equal); writing – review and editing (equal). **Qingji Zhang:** Data curation (equal); formal analysis (equal); investigation (equal); methodology (equal); supervision (equal); writing – review and editing (equal). **Xiaolong Wang:** Data curation (equal); formal analysis (equal); investigation (equal); methodology (equal). **Xianling Xiang:** Data curation (equal); formal analysis (equal); investigation (equal); methodology (equal). **Zhijun Gong:** Conceptualization (equal); formal analysis (equal); funding acquisition (equal); project administration (equal); supervision (equal); visualization (equal); writing – original draft (equal); writing – review and editing (equal). **Yongjiu Cai:** Conceptualization (equal); formal analysis (equal); funding acquisition (equal); project administration (equal); supervision (equal); visualization (equal); writing – original draft (equal); writing – review and editing (equal).

## CONFLICT OF INTEREST STATEMENT

We have no conflicts of interest to disclose.

## Supporting information


Data S1.

## Data Availability

The data that support the findings of this study are openly available for download from Dryad at: https://datadryad.org/stash/share/hm8jws9k9pla2n_glewimm7munvfpzjx5kg6oehen‐a.

## References

[ece311466-bib-0001] Aspin, T. W. H. , Khamis, K. , Matthews, T. J. , Milner, A. M. , O'Callaghan, M. J. , Trimmer, M. , Woodward, G. , & Ledger, M. E. (2019). Extreme drought pushes stream invertebrate communities over functional thresholds. Global Change Biology, 25, 230–244. 30346098 10.1111/gcb.14495 PMC7379955

[ece311466-bib-0002] Boersma, K. , Bogan, M. , Henrichs, B. , & Lytle, D. (2013). Invertebrate assemblages of pools in arid‐land streams have high functional redundancy and are resistant to severe drying. Freshwater Biology, 59, 491–501.

[ece311466-bib-0003] Bogan, M. T. , & Lytle, D. A. (2011). Severe drought drives novel community trajectories in desert stream pools. Freshwater Biology, 56, 2070–2081.

[ece311466-bib-0004] Bonada, N. , DolÉDec, S. , & Statzner, B. (2007). Taxonomic and biological trait differences of stream macroinvertebrate communities between mediterranean and temperate regions: Implications for future climatic scenarios. Global Change Biology, 13, 1658–1671.

[ece311466-bib-0005] Bond, N. R. , Lake, P. S. , & Arthington, A. H. (2008). The impacts of drought on freshwater ecosystems: An Australian perspective. Hydrobiologia, 600, 3–16.

[ece311466-bib-0006] Bonhomme, C. , Cereghino, R. , Jean‐François, C. , Compin, A. , Corbara, B. , Jassey, V. E. J. , Leflaive, J. , Farjalla, V. F. , Marino, N. A. C. , Rota, T. , Srivastava, D. S. , & Leroy, C. (2020). In situ resistance, not immigration, supports invertebrate community resilience to drought intensification in a neotropical ecosystem. The Journal of Animal Ecology, 90, 2015–2026. 33232512 10.1111/1365-2656.13392

[ece311466-bib-0007] Bruno, D. , Gutiérrez‐Cánovas, C. , Sánchez‐Fernández, D. , Velasco, J. , & Nilsson, C. (2016). Impacts of environmental filters on functional redundancy in riparian vegetation. The Journal of Animal Ecology, 53, 846–855.

[ece311466-bib-0008] Bujan, J. , Yanoviak, S. , & Kaspari, M. (2016). Desiccation resistance in tropical insects: Causes and mechanisms underlying variability in a Panama ant community. Ecology and Evolution, 6, 6282–6291. 27648242 10.1002/ece3.2355 PMC5016648

[ece311466-bib-0009] Cereghino, R. , Françoise, L. , Bonhomme, C. , Jean‐François, C. , Compin, A. , Corbara, B. , Jassey, V. E. J. , Leflaive, J. , Rota, T. , Farjalla, V. F. , & Leroy, C. (2020). Desiccation resistance traits predict freshwater invertebrate survival and community response to drought scenarios in a neotropical ecosystem. Ecological Indicators, 119, 106839.

[ece311466-bib-0010] Chaparro, G. , Horváth, Z. , & O'Farrell, I. (2018). Plankton metacommunities in floodplain wetlands under contrasting hydrological conditions. Freshwater Biology, 63, 380–391. 29937596 10.1111/fwb.13076 PMC5993336

[ece311466-bib-0011] Chapin, F. S. , & Díaz, S. (2020). Interactions between changing climate and biodiversity: Shaping humanity's future. Proceedings of the National Academy of Sciences of the United States of America, 117, 6295–6296. 32152122 10.1073/pnas.2001686117 PMC7104170

[ece311466-bib-0012] Chase, J. M. (2007). Drought mediates the importance of stochastic community assembly. Proceedings of the National Academy of Sciences of the United States of America, 104, 17430–17434. 17942690 10.1073/pnas.0704350104 PMC2077273

[ece311466-bib-0013] Chessman, B. (2014). Relationships between lotic macroinvertebrate traits and responses to extreme drought. Freshwater Biology, 60, 50–63.

[ece311466-bib-0014] Chester, E. T. , & Robson, B. J. (2011). Drought refuges, spatial scale and recolonisation by invertebrates in non‐perennial streams. Freshwater Biology, 56, 2094–2104.

[ece311466-bib-0015] Cornut, J. , Clivot, H. , Chauvet, E. , Elger, A. , Pagnout, C. , & Guérold, F. (2012). Effect of acidification on leaf litter decomposition in benthic and hyporheic zones of woodland streams. Water Research, 46, 6430–6444. 23069077 10.1016/j.watres.2012.09.023

[ece311466-bib-0016] Crabot, J. , Polasek, M. , Launay, B. , Pařil, P. , & Datry, T. (2021). Drying in newly intermittent rivers leads to higher variability of invertebrate communities. Freshwater Biology, 66, 730–744.

[ece311466-bib-0017] Datry, T. , Larned, S. , Fritz, K. , Bogan, M. , Wood, P. J. , Meyer, E. I. , & Lavoie, A. (2013). Broad‐scale patterns of invertebrate richness and community composition in temporary rivers: Effects of flow intermittence. Ecography, 37, 94–104.

[ece311466-bib-0018] Datry, T. , Moya, N. , Zubieta, J. , & Oberdorff, T. (2016). Determinants of local and regional communities in intermittent and perennial headwaters of the Bolivian Amazon. Freshwater Biology, 61, 1335–1349.

[ece311466-bib-0019] Dézerald, O. , Cereghino, R. , Corbara, B. , Dejean, A. , & Leroy, C. (2015). Functional trait responses of aquatic macroinvertebrates to simulated drought in a neotropical bromeliad ecosystem. Freshwater Biology, 60, 1917–1929.

[ece311466-bib-0020] Dézerald, O. , Leroy, C. , Corbara, B. , Dejean, A. , Talaga, S. , & Céréghino, R. (2016). Environmental drivers of invertebrate population dynamics in neotropical tank bromeliads. Freshwater Biology, 62, 229–242.

[ece311466-bib-0021] Diaz, R. J. , & Rosenberg, R. (2008). Spreading dead zones and consequences for marine ecosystems. Science, 321, 926–929. 18703733 10.1126/science.1156401

[ece311466-bib-0022] Díaz, S. , & Cabido, M. (2001). Vive la différence: Plant functional diversity matters to ecosystem processes. Trends in Ecology & Evolution, 16, 646–655. 10.1016/s0169-5347(01)02181-4 11369096

[ece311466-bib-0023] Dolédec, S. , Chessel, D. , ter Braak, C. J. F. , & Champely, S. (1996). Matching species traits to environmental variables: A new three‐table ordination method. Environmental and Ecological Statistics, 3, 143–166.

[ece311466-bib-0024] Dolédec, S. , Simon, L. , Blemus, J. , Rigal, A. , Robin, J. , & Mermillod‐Blondin, F. (2021). Multiple stressors shape invertebrate assemblages and reduce their trophic niche: A case study in a regulated stream. Science of the Total Environment, 773, 145061. 33940713 10.1016/j.scitotenv.2021.145061

[ece311466-bib-0025] Dong, R. , Wang, Y. , Lu, C. , Lei, G. , & Wen, L. (2021). The seasonality of macroinvertebrate β diversity along the gradient of hydrological connectivity in a dynamic river‐floodplain system. Ecological Indicators, 121, 107112.

[ece311466-bib-0026] Dray, S. , Choler, P. , Dolédec, S. , Peres‐Neto, P. R. , Thuiller, W. , Pavoine, S. , & ter Braak, C. J. F. (2014). Combining the fourth‐corner and the RLQ methods for assessing trait responses to environmental variation[J]. Ecology, 95, 14–21. 24649641 10.1890/13-0196.1

[ece311466-bib-0027] Epele, L. B. , Grech, M. G. , Williams‐Subiza, E. A. , Stenert, C. , McLean, K. , Greig, H. S. , Maltchik, L. , Pires, M. M. , Bird, M. S. , Boissezon, A. , Boix, D. , Demierre, E. , García, P. E. , Gascón, S. , Jeffries, M. , Kneitel, J. M. , Loskutova, O. , Manzo, L. M. , Mataloni, G. , … Batzer, D. P. (2022). Perils of life on the edge: Climatic threats to global diversity patterns of wetland macroinvertebrates. Science of the Total Environment, 820, 153052. 35063522 10.1016/j.scitotenv.2022.153052

[ece311466-bib-0028] Feng, L. , Hu, C. , Chen, X. , Cai, X. , Tian, L. , & Gan, W. (2012). Assessment of inundation changes of Poyang Lake using MODIS observations between 2000 and 2010. Remote Sensing of Environment, 121, 80–92.

[ece311466-bib-0029] Gamfeldt, L. , Hillebrand, H. , & Jonsson, P. R. (2008). Multiple functions increase the importance of biodiversity for overall ecosystem functioning. Ecology, 89, 1223–1231. 18543617 10.1890/06-2091.1

[ece311466-bib-0030] Gross, N. , Le Bagousse‐Pinguet, Y. , Liancourt, P. , et al. (2017). Functional trait diversity maximizes ecosystem multifunctionality. Nature Ecology & Evolution, 1, 132. 28812705 10.1038/s41559-017-0132

[ece311466-bib-0031] Guan, Q. , Wu, H. , Lu, K. , Lu, X. , & Batzer, D. P. (2017). Longitudinal and lateral variation in snail assemblages along a floodplain continuum. Hydrobiologia, 792, 345–356.

[ece311466-bib-0032] Gusmao, J. , Brauko, K. , Eriksson, B. , & Lana, P. (2016). Functional diversity of macrobenthic assemblages decreases in response to sewage discharges. Ecological Indicators, 66, 65–75.

[ece311466-bib-0033] Habagil, M. (2019). Global diversity and biogeography of bacterial communities in wastewater treatment plants. Nature Microbiology, 4, 1183–1195. 10.1038/s41564-019-0426-5 31086312

[ece311466-bib-0034] Heino, J. , Melo, A. , & Bini, L. (2014). Reconceptualising the beta diversity‐environmental heterogeneity relationship in running water systems. Freshwater Biology, 60, 223–235.

[ece311466-bib-0035] Heino, J. , Melo, A. S. , Siqueira, T. , Soininen, J. , Valanko, S. , & Bini, L. M. (2015). Metacommunity organisation, spatial extent and dispersal in aquatic systems: Patterns, processes and prospects. Freshwater Biology, 60, 845–869.

[ece311466-bib-0036] Herbst, D. , Cooper, S. , Medhurst, R. , Wiseman, S. W. , & Hunsaker, C. T. (2019). Drought ecohydrology alters the structure and function of benthic invertebrate communities in mountain streams[J]. Freshwater Biology, 64, 886–902.

[ece311466-bib-0037] Hoverman, J. T. , Davis, C. J. , Werner, E. E. , Skelly, D. K. , Relyea, R. A. , & Yurewicz, K. L. (2011). Environmental gradients and the structure of freshwater snail communities. Ecography, 34, 1049–1058.

[ece311466-bib-0038] Humphries, P. , & Baldwin, D. S. (2003). Drought and aquatic ecosystems: An introduction. Freshwater Biology, 48, 1141–1146.

[ece311466-bib-0039] Jaeger, K. , Olden, J. , & Pelland, N. (2014). Climate change poised to threaten hydrologic connectivity and endemic fishes in dryland streams. Proceedings of the National Academy of Sciences of the United States of America, 111, 13894–13899. 25136090 10.1073/pnas.1320890111 PMC4183275

[ece311466-bib-0040] Jun, X. , Qiting, Z. , & Chunhui, H. (2018). Disciplinary system and development strategy for eco‐hydrology. Advances in Earth Science, 33, 665.

[ece311466-bib-0041] Kleyer, M. , Dray, S. , Bello, F. , Lepš, J. , Pakeman, R. J. , Strauss, B. , Thuiller, W. , & Lavorel, S. (2012). Assessing species and community functional responses to environmental gradients: Which multivariate methods? Journal of Vegetation Science, 23, 805–821.

[ece311466-bib-0042] Knox, R. L. , Wohl, E. E. , & Morrison, R. R. (2022). Levees don't protect, they disconnect: A critical review of how artificial levees impact floodplain functions. Science of the Total Environment, 837, 155773. 35537517 10.1016/j.scitotenv.2022.155773

[ece311466-bib-0043] Koudenoukpo, Z. C. , Odountan, O. H. , Agboho, P. A. , Dalu, T. , van Bocxlaer, B. , Janssens de Bistoven, L. , Chikou, A. , & Backeljau, T. (2021). Using self–organizing maps and machine learning models to assess mollusc community structure in relation to physicochemical variables in a West Africa river–estuary system. Ecological Indicators, 126, 107706.

[ece311466-bib-0044] Larned, S. , Datry, T. , & Robinson, C. (2007). Invertebrate and microbial responses to inundation in an ephemeral river reach in New Zealand: Effects of preceding dry periods[J]. Aquatic Sciences, 69, 554–567.

[ece311466-bib-0045] Ledger, M. , & Hildrew, A. (2005). The ecology of acidification and recovery: Changes in herbivore‐algal food web linkages across a stream pH gradient. Environmental Pollution, 137, 103–118. 15944043 10.1016/j.envpol.2004.12.024

[ece311466-bib-0046] Ledger, M. E. , Brown, L. E. , Edwards, F. K. , Milner, A. M. , & Woodward, G. (2013). Drought alters the structure and functioning of complex food webs[J]. Nature Climate Change, 3, 223–227.

[ece311466-bib-0047] Legendre, P. , & Condit, R. (2019). Spatial and temporal analysis of beta diversity in the Barro Colorado Island forest dynamics plot, Panama. Forest Ecosystems, 6, 7.

[ece311466-bib-0048] Leigh, C. (2013). Dry‐season changes in macroinvertebrate assemblages of highly seasonal rivers: Responses to low flow, no flow and antecedent hydrology. Hydrobiologia, 703, 95–112.

[ece311466-bib-0049] Li, X. , Ye, X. , Yuan, C. , & Xu, C. (2023). Can water release from local reservoirs cope with the droughts of downstream lake in a large river‐lake system? Journal of Hydrology, 625, 130172.

[ece311466-bib-0050] Lopes‐Lima, M. , Burlakova, L. E. , Karatayev, A. Y. , Mehler, K. , Seddon, M. , & Sousa, R. (2018). Conservation of freshwater bivalves at the global scale: Diversity, threats and research needs. Hydrobiologia, 810, 1–14.

[ece311466-bib-0051] Lyu, Z. Z. , Gao, H. , Gao, R. , & Ding, T. (2023). Extreme characteristics and causes of the drought event in the whole Yangtze River basin in the midsummer of 2022. Advances in Climate Change Research, 146, 50–59.

[ece311466-bib-0052] Magurran, A. E. (2021). Measuring biological diversity. Current Biology, 31, R1174–R1177. 34637726 10.1016/j.cub.2021.07.049

[ece311466-bib-0053] Mammola, S. , Carmona, C. P. , Guillerme, T. , & Cardoso, P. (2021). Concepts and applications in functional diversity. Functional Ecology, 35, 1869–1885.

[ece311466-bib-0054] Mason, N. W. H. , Mouillot, D. , Lee, W. G. , & Wilson, J. B. (2005). Functional richness, functional evenness and functional divergence: The primary components of functional diversity. Oikos, 111, 112–118.

[ece311466-bib-0055] Meena, D. K. , Lianthuamluaia, L. , Mishal, P. , Swain, H. S. , Naskar, B. K. , Saha, S. , Sandhya, K. M. , Kumari, S. , Tayung, T. , Sarkar, U. K. , & das, B. K. (2019). Assemblage patterns and community structure of macro‐zoobenthos and temporal dynamics of eco‐physiological indices of two wetlands, in lower gangetic plains under varying ecological regimes: A tool for wetland management. Ecological Engineering, 130, 1–10.

[ece311466-bib-0056] Miller, A. , Brown, J. , Enderling, H. , Basanta, D. , & Whelan, C. J. (2021). The evolutionary ecology of dormancy in nature and in cancer. Frontiers in Ecology and Evolution, 9, 676802.

[ece311466-bib-0057] Mouillot, D. , Villéger, S. , Parravicini, V. , Kulbicki, M. , Arias‐González, J. E. , Bender, M. , Chabanet, P. , Floeter, S. R. , Friedlander, A. , Vigliola, L. , & Bellwood, D. R. (2014). Functional over‐redundancy and high functional vulnerability in global fish faunas on tropical reefs. Proceedings of the National Academy of Sciences of the United States of America, 111, 13757–13762. 25225388 10.1073/pnas.1317625111 PMC4183327

[ece311466-bib-0058] Mu, S. , Li, B. , Yao, J. , Yang, G. , Wan, R. , & Xu, X. (2020). Monitoring the spatio‐temporal dynamics of the wetland vegetation in Poyang Lake by Landsat and MODIS observations. Science of the Total Environment, 725, 138096. 32302824 10.1016/j.scitotenv.2020.138096

[ece311466-bib-0059] Nasi, F. , Nordström, M. , Bonsdorff, E. , Auriemma, R. , Cibic, T. , & Del Negro, P. (2018). Functional biodiversity of marine soft‐sediment polychaetes from two mediterranean coastal areas in relation to environmental stress. Marine Environmental Research, 137, 121–132. 29551408 10.1016/j.marenvres.2018.03.002

[ece311466-bib-0060] Nelson, D. , Busch, M. H. , Kopp, D. A. , & Allen, D. C. (2021). Energy pathways modulate the resilience of stream invertebrate communities to drought. The Journal of Animal Ecology, 90, 2053–2064. 33782972 10.1111/1365-2656.13490

[ece311466-bib-0061] Nieto, C. , Ovando, X. M. C. , Loyola, R. , Izquierdo, A. , Romero, F. , Molineri, C. , Rodríguez, J. , Rueda Martín, P. , Fernández, H. , Manzo, V. , & Miranda, M. J. (2017). The role of macroinvertebrates for conservation of freshwater systems. Ecology and Evolution, 7, 5502–5513. 28770086 10.1002/ece3.3101 PMC5528230

[ece311466-bib-0062] Palmer, M. , & Ruhi, A. (2019). Linkages between flow regime, biota, and ecosystem processes: Implications for river restoration. Science, 365, eaaw2087. 31604208 10.1126/science.aaw2087

[ece311466-bib-0063] Petchey, O. , Evans, K. , Fishburn, I. , & Gaston, K. (2007). Low functional diversity and no redundancy in British avian assemblages. The Journal of Animal Ecology, 76, 977–985. 17714276 10.1111/j.1365-2656.2007.01271.x

[ece311466-bib-0064] Philpott, S. , Pardee, G. , & Gonthier, D. (2012). Cryptic biodiversity effects: Importance of functional redundancy revealed through addition of food web complexity. Ecology, 93, 992–1001. 22764486 10.1890/11-1431.1

[ece311466-bib-0065] Poff, N. L. , & Zimmerman, J. K. H. (2010). Ecological responses to altered flow regimes: A literature review to inform the science and management of environmental flows. Freshwater Biology, 55, 194–205.

[ece311466-bib-0066] Poznańska, M. , Kakareko, T. , Gulanicz, T. , Jermacz, Ł. , & Kobak, J. (2015). Life on the edge: Survival and behavioural responses of freshwater gill‐breathing snails to declining water level and substratum drying. Freshwater Biology, 60, 2379–2391.

[ece311466-bib-0067] Prada‐Salcedo, L. D. , Wambsganss, J. , Bauhus, J. , Buscot, F. , & Goldmann, K. (2021). Low root functional dispersion enhances functionality of plant growth by influencing bacterial activities in European forest soils. Environmental Microbiology, 23, 1889–1906. 32959469 10.1111/1462-2920.15244

[ece311466-bib-0068] Rice, E. W. , Bridgewater, L. , & Association A P H . (2012). Standard methods for the examination of water and wastewater[M].L 10. American Public Health Association.

[ece311466-bib-0069] Ricotta, C. , de Bello, F. , Moretti, M. , Caccianiga, M. , Cerabolini, B. E. L. , & Pavoine, S. (2016). Measuring the functional redundancy of biological communities: A quantitative guide. Methods in Ecology and Evolution, 7, 1386–1395.

[ece311466-bib-0070] Robson, B. J. , Chester, E. T. , & Austin, C. M. (2011). Why life history information matters: Drought refuges and macroinvertebrate persistence in non‐perennial streams subject to a drier climate. Marine and Freshwater Research, 62, 801–810.

[ece311466-bib-0071] Rosenfeld, J. (2002). Functional redundancy in ecology and conservation. Oikos, 98, 156–162.

[ece311466-bib-0072] Ruhi, A. , Datry, T. , & Sabo, J. L. (2017). Interpreting beta‐diversity components over time to conserve metacommunities in highly dynamic ecosystems. Conservation Biology, 31, 1459–1468. 28188969 10.1111/cobi.12906

[ece311466-bib-0073] Socolar, J. B. , Gilroy, J. J. , Kunin, W. E. , & Edwards, D. P. (2016). How should Beta‐diversity inform biodiversity conservation? Trends in Ecology & Evolution, 31, 67–80. 26701706 10.1016/j.tree.2015.11.005

[ece311466-bib-0074] Strong, E. E. , Gargominy, O. , Ponder, W. F. , & Bouchet, P. (2008). Global diversity of gastropods (Gastropoda; Mollusca) in freshwater. Hydrobiologia, 595, 149–166.

[ece311466-bib-0075] Trenberth, K. E. , Dai, A. , Schrier, G. , Trenberth, K. E. , Mann, M. E. , & Abraham, J. P. (2014). Increasing ocean stratification over the past half‐century. Nature Climate Change, 4, 17–22.

[ece311466-bib-0076] Trzcinski, M. , Srivastava, D. , Corbara, B. , Dézerald, O. , Leroy, C. , Carrias, J.‐F. , Dejean, A. , & Céréghino, R. (2016). The effects of food‐web structure on ecosystem function exceeds those of precipitation. The Journal of Animal Ecology, 85, 1147–1160. 27120013 10.1111/1365-2656.12538

[ece311466-bib-0077] Vander Vorste, R. , Corti, R. , Sagouis, A. , & Datry, T. (2015). Invertebrate communities in gravel‐bed, braided rivers are highly resilient to flow intermittence. Freshwater Biology, 35, 164–177.

[ece311466-bib-0078] Vander Vorste, R. , Malard, F. , & Datry, T. (2015). Is drift the primary process promoting the resilience of river invertebrate communities? A manipulative field experiment in an intermittent alluvial river. Freshwater Biology, 61, 1276–1292.

[ece311466-bib-0079] Vaughn, C. C. (2018). Ecosystem services provided by freshwater mussels. Hydrobiologia, 810, 15–27.

[ece311466-bib-0080] Vaughn, C. C. , Atkinson, C. L. , & Julian, J. P. (2015). Drought‐induced changes in flow regimes lead to long‐term losses in mussel‐provided ecosystem services. Ecology and Evolution, 5, 1291–1305. 25859334 10.1002/ece3.1442 PMC4377272

[ece311466-bib-0081] Verdonschot, R. , Oosten‐Siedlecka, A. , ter Braak, C. , & Verdonschot, P. (2014). Macroinvertebrate survival during cessation of flow and streambed drying in a lowland stream. Freshwater Biology, 60, 282–296.

[ece311466-bib-0082] Villéger, S. , Mason, N. W. H. , & Mouillot, D. (2008). New multidimensional functional diversity indices for a multifaced framework in functional ecology. Ecology, 89, 2290–2301. 18724739 10.1890/07-1206.1

[ece311466-bib-0083] Walker, D. W. , & Van Loon, A. F. (2023). Droughts are coming on faster. Science, 380, 130–132. 37053333 10.1126/science.adh3097

[ece311466-bib-0084] Wang, J. , Ding, C. , Tao, J. , Jiang, X. , Heino, J. , Ding, L. , Su, W. , Chen, M. , Zhang, K. , & He, D. (2021). Damming affects riverine macroinvertebrate metacommunity dynamics: Insights from taxonomic and functional beta diversity[J]. Science of the Total Environment, 763, 142945. 33127148 10.1016/j.scitotenv.2020.142945

[ece311466-bib-0085] Wu, H. , Guan, Q. , Lu, X. , & Batzer, D. P. (2017). Snail (Mollusca: Gastropoda) assemblages as indicators of ecological condition in freshwater wetlands of northeastern China. Ecological Indicators, 75, 203–209.

[ece311466-bib-0086] Yuan, X. , Wang, Y. , Ji, P. , Wu, P. , Sheffield, J. , & Otkin, J. A. (2023). A global transition to flash droughts under climate change. Science, 380, 187–191. 37053316 10.1126/science.abn6301

[ece311466-bib-0087] Zhang, J. , An, S. , & Leng, X. (2020). Status of wetland research in China. Marine and Freshwater Research, 71, 1572.

[ece311466-bib-0088] Zhang, L. , Pan, B. , Jiang, X. , Wang, H. , Lu, Y. , Lu, Y. , & Li, R. (2020). Responses of the macroinvertebrate taxonomic distinctness indices of lake fauna to human disturbances in the middle and lower reaches of the Yangtze river. Ecological Indicators, 110, 105952.

