# Supplementary material for: Effects of different hydrological conditions on the taxonomic structure and functional traits of mollusk communities in a large floodplain wetland
Source: Ecol Evol. 2024 May 26;14(5):e11466. doi: 10.1002/ece3.11466 (PMC11128460; doi:10.1002/ece3.11466)
Supplement: Supplementary file 1 — Data S1. [file ECE3-14-e11466-s001.docx]

# Effects of different hydrological conditions on the taxonomic structure and functional traits of mollusk communities in a large floodplain wetland

# Supporting information


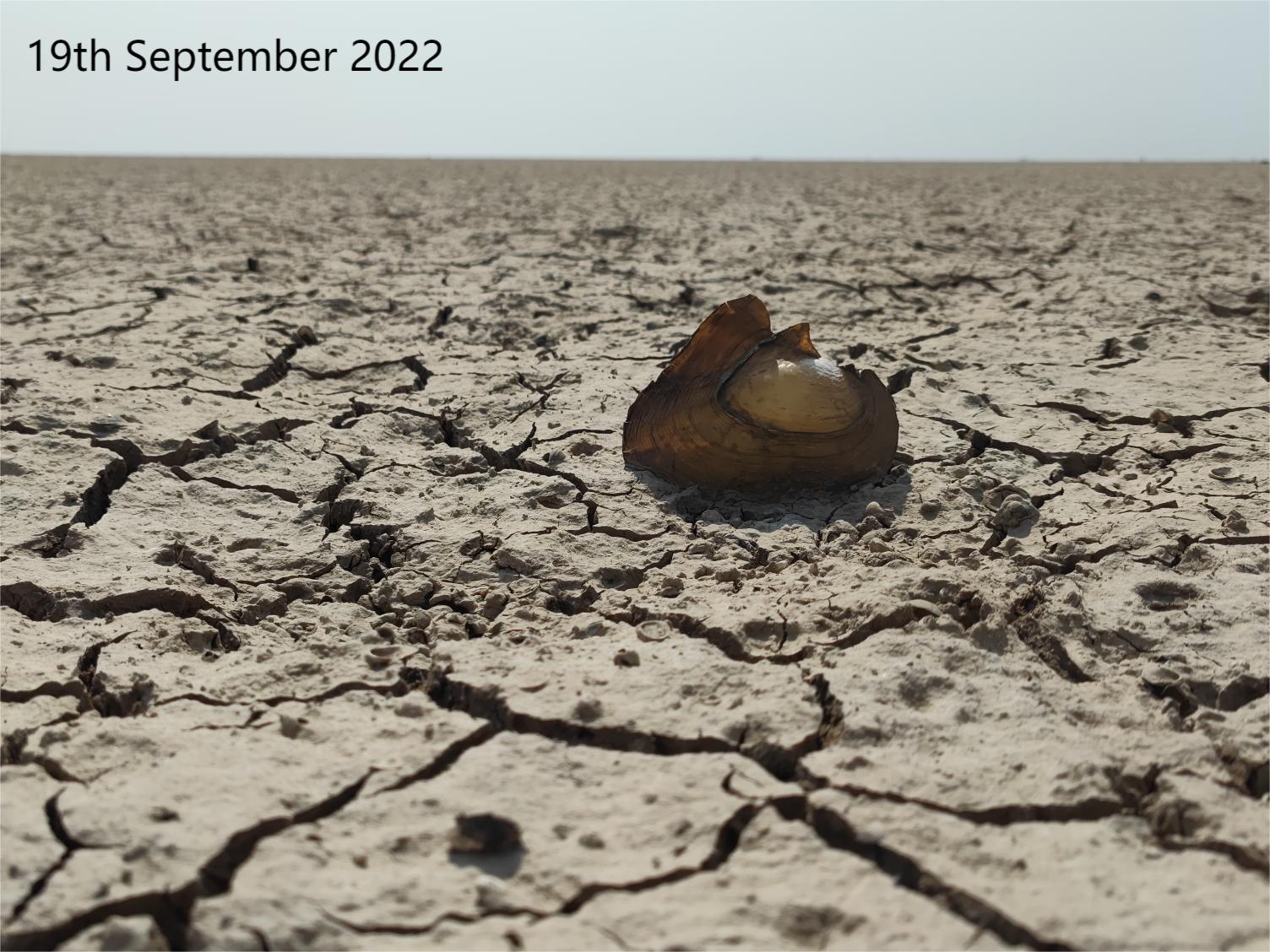

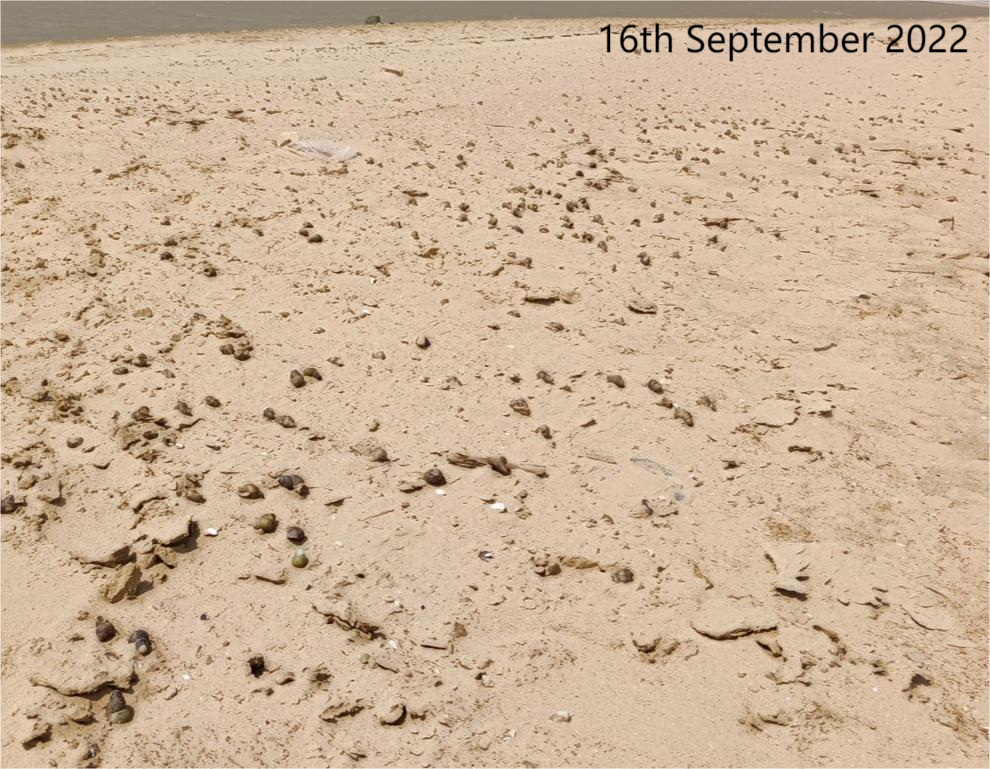


**Fig. S1** Habitat photos in sub-lakes. Sudden droughts (high temperatures, severe droughts) cause the death of large numbers of mollusks.


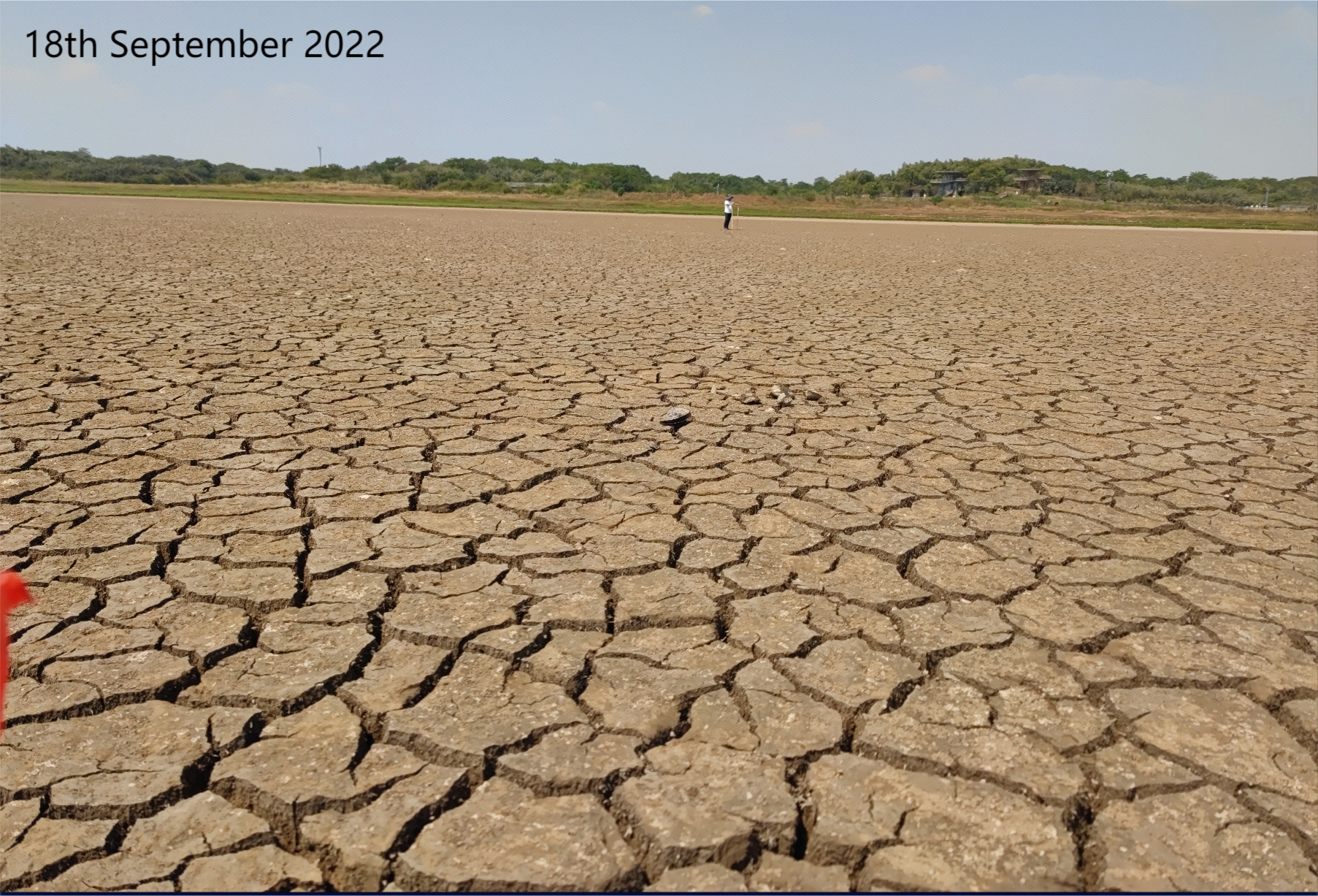


**Fig. S2** Photographs of lake bottom habitats in Changhuchi. The sudden drought caused Changhuchi to completely dry up for the first time in nearly 70 years and created numerous cracks in the bottom of the lake. No surviving mollusks were found during this survey.


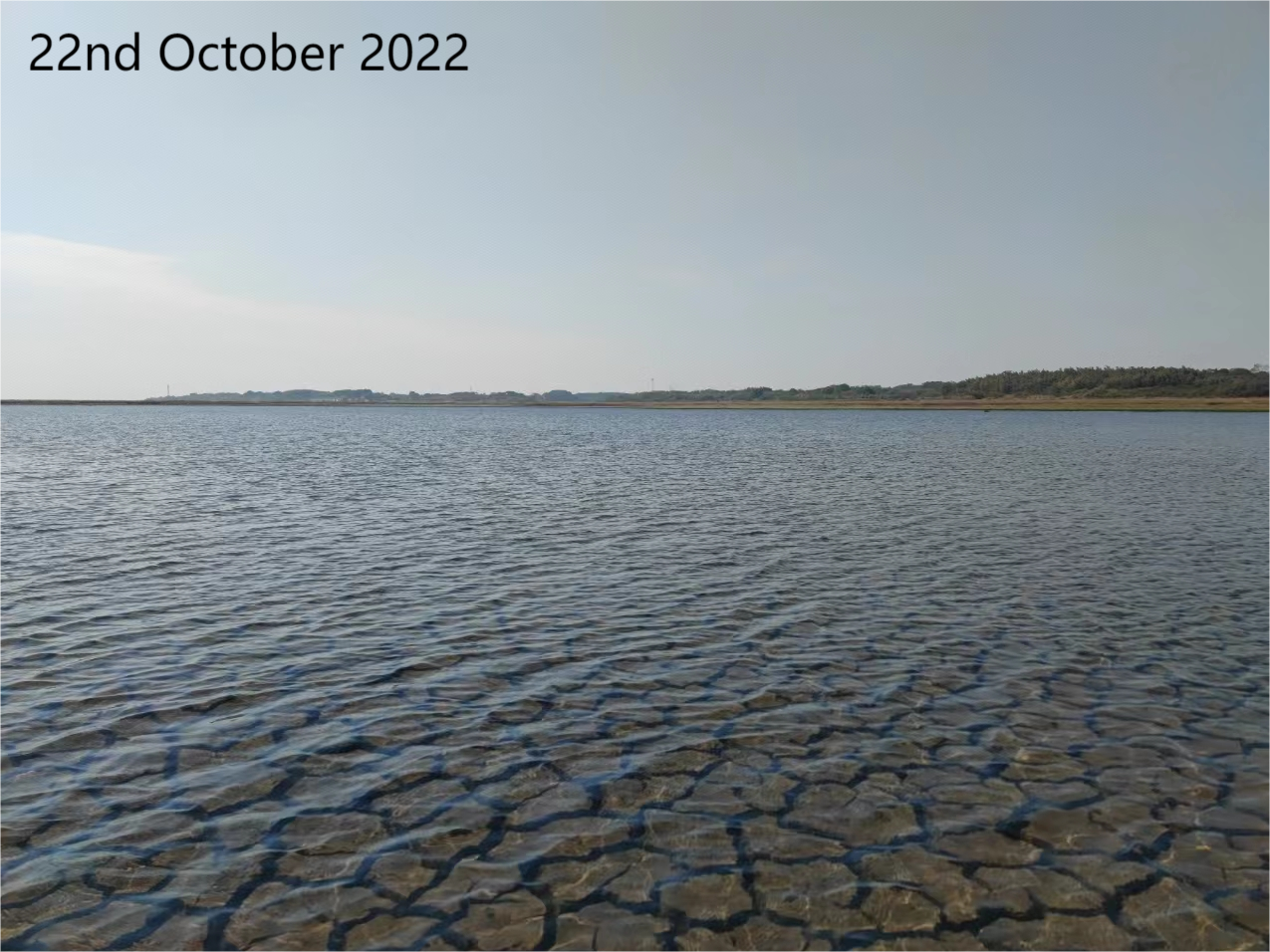


**Fig. S3** Photographs of lake bottom habitats in Changhuchi. Pumping of water from the Gan River into Changhuchi on 29 September 2022 and maintaining a water depth of not less than 30 cm thereafter.

**Table S1** Sampling information for the different sampling occasions

| Sampling period | Sampling sites | Water level (m)（Xingzi station） | Hydrology condition |
| --- | --- | --- | --- |
| Jul-14 | Meixihu 1# | 18.8 | Wet season（Pre1） |
|  | Meixihu 2# |  |  |
|  | Meixihu 3# |  |  |
|  | Zhonghuchi 1# |  |  |
|  | Zhonghuchi 2# |  |  |
|  | Xianghu 1# |  |  |
|  | Xianghu 2# |  |  |
|  | Xianghu 3# |  |  |
|  | Changhuchi 1# |  |  |
|  | Changhuchi 2# |  |  |
|  | Changhuchi 3# |  |  |
|  | Shahu 1# |  |  |
|  | Shahu 2# |  |  |
|  | Shahu 3# |  |  |
|  | Shahu 4# |  |  |
|  | Dachahu 1# |  |  |
|  | Dachahu 2# |  |  |
|  | Dachahu 3# |  |  |
|  | Dachahu 4# |  |  |
|  | Zhushihu 1# |  |  |
|  | Zhushihu 2# |  |  |
|  | Zhushihu 3# |  |  |
|  | Dahuchi 1# |  |  |
|  | Dahuchi 2# |  |  |
|  | Dahuchi 3# |  |  |
|  | Dahuchi 4# |  |  |
|  | Dahuchi 5# |  |  |
| Dec-14 | Meixihu 1# | 8 | normal drought（Pre2） |
|  | Meixihu 2# |  |  |
|  | Meixihu 3# |  |  |
|  | Zhonghuchi 1# |  |  |
|  | Zhonghuchi 2# |  |  |
|  | Zhonghuchi 3# |  |  |
|  | Xianghu 1# |  |  |
|  | Xianghu 2# |  |  |
|  | Xianghu 3# |  |  |
|  | Changhuchi 1# |  |  |
|  | Changhuchi 2# |  |  |
|  | Changhuchi 3# |  |  |
|  | Shahu 1# |  |  |
|  | Shahu 2# |  |  |
|  | Shahu 3# |  |  |
|  | Dachahu 1# |  |  |
|  | Dachahu 2# |  |  |
|  | Zhushihu 1# |  |  |
|  | Zhushihu 2# |  |  |
|  | Zhushihu 3# |  |  |
|  | Dahuchi 1# |  |  |
|  | Dahuchi 2# |  |  |
|  | Dahuchi 3# |  |  |
|  | Dahuchi 4# |  |  |
| Sep-22 | Meixihu 1# | 7 | flash drought（FD） |
|  | Zhonghuchi 1# |  |  |
|  | Shahu 1# |  |  |
|  | Dachahu 1# |  |  |
|  | Zhushihu 1# |  |  |
|  | Dahuchi 1# |  |  |
| Oct-22 | Changhuchi 4# | 7 | rewetting 1 month (Pos1) |
|  | Changhuchi 5# |  |  |
|  | Changhuchi 6# |  |  |
| Nov-22 | Changhuchi 4# | 7 | rewetting 2 month (Pos2) |
|  | Changhuchi 5# |  |  |
|  | Changhuchi 6# |  |  |
| Feb-23 | Changhuchi 4# | 7 | rewetting 5 month (Pos3) |
|  | Changhuchi 5# |  |  |
|  | Changhuchi 6# |  |  |

**Table S2** Relative densities, relative biomass, frequency of occurrence and dominance of species in the sub-lakes

| Time | |  | *C fluminea* | *L fortunei* | *A elliptica* | *A pacifica* | *A lanceolata* | *S lampreyanus* | *Unio douglasiae* | *B aeruginosa* | *S cancelata* | *P eximius* | *P striatulus* | *P sinensis* | *A longicornis* | *P anomalospiralis* | *R swinhoei* | *C chinensis* | *B Moellendorff* |
| --- | --- | --- | --- | --- | --- | --- | --- | --- | --- | --- | --- | --- | --- | --- | --- | --- | --- | --- | --- |
| Pre1 (wet season) | 2014/6 | Relative density(%) | 7.17% | 0.33% | 0.00% | 0.44% | 0.17% | 0.00% | 0.00% | 34.64% | 0.55% | 3.86% | 11.47% | 8.22% | 25.98% | 1.38% | 0.00 | 0.00 | 0.06 |
|  |  | Relative biomass(%) | 30.24% | 0.96% | 0.00% | 0.57% | 3.67% | 0.00% | 0.00% | 51.65% | 0.12% | 2.73% | 2.34% | 2.15% | 4.09% | 0.24% | 0.00 | 0.00 | 0.01 |
|  |  | Frequency(%) | 53.85% | 3.85% | 0.00% | 7.69% | 3.85% | 0.00% | 0.00% | 96.15% | 3.85% | 30.77% | 46.15% | 15.38% | 57.69% | 7.69% | 0.00 | 0.00 | 0.27 |
|  |  | Dominance | 2014.52 | 4.97 | 0.00 | 7.78 | 14.73 | 0.00 | 0.00 | 8297.37 | 2.57 | 202.93 | 637.46 | 159.55 | 1734.94 | 12.45 | 0.00 | 0.00 | 189.09 |
| Pre2 (dry season) | 2014/6 | Relative density(%) | 3.05% | 0.00% | 0.76% | 0.00% | 0.00% | 0.11% | 0.80% | 40.65% | 0.00% | 5.20% | 13.96% | 19.77% | 15.42% | 0.14% | 0.00 | 0.00 | 0.00 |
|  |  | Relative biomass(%) | 3.01% | 0.00% | 48.74% | 0.00% | 0.00% | 2.28% | 4.30% | 33.46% | 0.00% | 3.08% | 1.72% | 2.29% | 1.11% | 0.01% | 0.00 | 0.00 | 0.00 |
|  |  | Frequency(%) | 43.48% | 0.00% | 17.39% | 0.00% | 0.00% | 4.35% | 17.39% | 78.26% | 0.00% | 65.22% | 47.83% | 47.83% | 56.52% | 4.35% | 0.04 | 0.00 | 0.00 |
|  |  | Dominance | 6054.03 | 0.00 | 19798.49 | 0.00 | 0.00 | 238.49 | 2040.09 | 133405.40 | 0.00 | 12421.71 | 17238.89 | 24273.69 | 21490.42 | 14.83 | 15.14 | 0.00 | 0.00 |
| FD (flash drought) | 2022/9 | Relative density(%) | 0.00% | 0.00% | 0.00% | 0.00% | 0.00% | 0.00% | 0.00% | 67.06% | 0.00% | 16.47% | 0.00% | 1.18% | 0.00% | 0.00% | 0.12 | 0.04 | 0.00 |
|  |  | Relative biomass(%) | 0.00% | 0.00% | 0.00% | 0.00% | 0.00% | 0.00% | 0.00% | 79.26% | 0.00% | 7.39% | 0.00% | 0.28% | 0.00% | 0.00% | 0.01 | 0.12 | 0.00 |
|  |  | Frequency(%) | 0.00% | 0.00% | 0.00% | 0.00% | 0.00% | 0.00% | 0.00% | 100.00% | 0.00% | 33.33% | 0.00% | 16.67% | 0.00% | 0.00% | 0.33 | 0.17 | 0.00 |
|  |  | Dominance | 0.00 | 0.00 | 0.00 | 0.00 | 0.00 | 0.00 | 0.00 | 14631.90 | 0.00 | 795.20 | 0.00 | 24.31 | 0.00 | 0.00 | 420.58 | 262.48 | 0.00 |

**Table S3** Relative densities, relative biomass, frequency of occurrence and dominance of species in the Changhuchi

|  | Time | |  | *C fluminea* | *A elliptica* | *U douglasiae* | *B aeruginosa* | *P eximius* | *P striatulus* | *A longicornis* | *C chinensis* | *B Moellendorff* |
| --- | --- | --- | --- | --- | --- | --- | --- | --- | --- | --- | --- | --- |
| Pre1 | wet season | 2014/7 | Relative density(%) | 0.02 | 0.00 | 0.00 | 0.67 | 0.08 | 0.10 | 0.04 | 0.00 | 0.10 |
|  |  |  | Relative biomass(%) | 0.07 | 0.00 | 0.00 | 0.85 | 0.04 | 0.01 | 0.01 | 0.00 | 0.01 |
|  |  |  | Frequency(%) | 0.33 | 0.00 | 0.00 | 1.00 | 1.00 | 0.67 | 0.67 | 0.00 | 0.67 |
|  |  |  | Dominance | 290.62 | 0.00 | 0.00 | 15260.43 | 1181.82 | 739.18 | 312.86 | 0.00 | 738.55 |
| Pre2 | dry season | 2014/12 | Relative density(%) | 0.14 | 0.07 | 0.29 | 0.07 | 0.07 | 0.00 | 0.36 | 0.00 | 0.00 |
|  |  |  | Relative biomass(%) | 0.00 | 0.37 | 0.59 | 0.02 | 0.01 | 0.00 | 0.01 | 0.00 | 0.00 |
|  |  |  | Frequency(%) | 0.67 | 0.33 | 0.67 | 0.33 | 0.33 | 0.00 | 0.33 | 0.00 | 0.00 |
|  |  |  | Dominance | 4284.34 | 52422.92 | 174342.28 | 4430.07 | 1923.74 | 0.00 | 6716.69 | 0.00 | 0.00 |
| Pos1 | rewet 1 month | 2022/10 | Relative density(%) | 0.00 | 0.00 | 0.00 | 0.44 | 0.44 | 0.00 | 0.00 | 0.11 | 0.00 |
|  |  |  | Relative biomass(%) | 0.00 | 0.00 | 0.00 | 0.47 | 0.20 | 0.00 | 0.00 | 0.34 | 0.00 |
|  |  |  | Frequency(%) | 0.00 | 0.00 | 0.00 | 1.00 | 1.00 | 0.00 | 0.00 | 0.33 | 0.00 |
|  |  |  | Dominance | 0.00 | 0.00 | 0.00 | 9094.58 | 6427.92 | 0.00 | 0.00 | 1492.50 | 0.00 |
| Pos2 | rewet 2 month | 2022/11 | Relative density(%) | 0.00 | 0.00 | 0.00 | 0.69 | 0.31 | 0.00 | 0.00 | 0.00 | 0.00 |
|  |  |  | Relative biomass(%) | 0.00 | 0.00 | 0.00 | 0.84 | 0.16 | 0.00 | 0.00 | 0.00 | 0.00 |
|  |  |  | Frequency(%) | 0.00 | 0.00 | 0.00 | 1.00 | 0.33 | 0.00 | 0.00 | 0.00 | 0.00 |
|  |  |  | Dominance | 0.00 | 0.00 | 0.00 | 15251.04 | 1582.99 | 0.00 | 0.00 | 0.00 | 0.00 |
| Pos3 | rewet 5 month | 2023/2 | Relative density(%) | 0.00 | 0.00 | 0.00 | 0.92 | 0.08 | 0.00 | 0.00 | 0.00 | 0.00 |
|  |  |  | Relative biomass(%) | 0.00 | 0.00 | 0.00 | 0.96 | 0.04 | 0.00 | 0.00 | 0.00 | 0.00 |
|  |  |  | Frequency(%) | 0.00 | 0.00 | 0.00 | 0.67 | 0.33 | 0.00 | 0.00 | 0.00 | 0.00 |
|  |  |  | Dominance | 0.00 | 0.00 | 0.00 | 12528.92 | 402.21 | 0.00 | 0.00 | 0.00 | 0.00 |

**Table S4** Functional traits, categories, and abbreviations in macrobenthos

| Functional traits | Trait modalities | Abbrev |
| --- | --- | --- |
| Maximal size | ≤0.25cm | B1 |
|  | 0.25-0.5 | B2 |
|  | 0.5-1 | B3 |
|  | 1-2 | B4 |
|  | 2-4 | B5 |
|  | 4-8 | B6 |
|  | >8 | B7 |
| Life cycle duration | ≤1 year | LI1 |
|  | >1 year | LI2 |
| Aquatic stages | egg | A1 |
|  | larva | A2 |
|  | pupa | A3 |
|  | adult | A4 |
| Reproduction | ovoviviparity | RP1 |
|  | isolated eggs, free | RP2 |
|  | isolated eggs,cemented | RP3 |
|  | clutches,cemented or fixed | RP4 |
|  | clutches,free | RP5 |
|  | clutches,in vegetation | RP6 |
|  | clutches,terrestrial | RP7 |
|  | asexual reproduction | RP8 |
| Dissemination | aquatic passive | D1 |
|  | aquatic active | D2 |
|  | aerial passive | D3 |
|  | aerial active | D4 |
| Resistance form | eggs,statoblasts | RF1 |
|  | cocoons | RF2 |
|  | cells against desiccation | RF3 |
|  | diapause or dormancy | RF4 |
|  | none | RF5 |
| Respiration | tegument | RS1 |
|  | gill | RS2 |
|  | plastron | RS3 |
|  | spiracle(aerial) | RS4 |
|  | hydrostatic vesicle(aerial) | RS5 |
| Locomotion and substrate relation | flier | LO1 |
|  | surface swimmer | LO2 |
|  | full water swimmer | LO3 |
|  | crawler | LO4 |
|  | burrower(epibenthic) | LO5 |
|  | interstitial(epibenthic) | LO6 |
|  | temporarily attached | LO7 |
|  | permanently attached | LO8 |
| Food | fine sediment + microorganisms | FO1 |
|  | detritus<1mm | FO2 |
|  | plant detritus>1mm | FO3 |
|  | living microphytes | FO4 |
|  | living macrophytes | FO5 |
|  | dead animal>1mm | FO6 |
|  | living microinvertebrates | FO7 |
|  | living macroinvertebrates | FO8 |
|  | vertebrates | FO9 |
| Feeding habits | absorber | FE1 |
|  | deposit feeder | FE2 |
|  | shredder | FE3 |
|  | scraper | FE4 |
|  | filter feeder | FE5 |
|  | piercer (plants or animals) | FE6 |
|  | predator (carver/engulfer/swallower) | FE7 |
|  | parasite | FE8 |

**Table S5** Abbreviations in macrobenthos

| Species | Abbrev |
| --- | --- |
| *Corbicula fluminea* | SP1 |
| *Limnoperna fortunei* | SP2 |
| *Anodonta woodiana elliptica* | SP3 |
| *Anodonta woodia. pacifica* | SP4 |
| *Arconaia lanceolata* | SP5 |
| *Schistodesmus lampreyanus* | SP6 |
| *Unio douglasiae* | SP7 |
| *Bellamya aeruginosa* | SP8 |
| *Semisulcospira cancelata* | SP9 |
| *Semisulcospira libertina* | SP10 |
| *Parafossarulus eximius* | SP11 |
| *Parafossarulus striatulus* | SP12 |
| *Parafossarulus sinensis* | SP13 |
| *Alocinma longicornis* | SP14 |
| *Parafossarulus anomalospiralis* | SP15 |
| *Radix swinhoei* | SP16 |
| *Cipangopaludina chinensis* | SP17 |
| *Bithynia fuchsiana Moellendorff* | SP18 |

**Table S6** One-way ANOVA summary of environmental variables at different times in Sub-lakes. P-values: the significance of differences in environmental factors across time.

| Sub-lakes | | | | | |
| --- | --- | --- | --- | --- | --- |
| Environment variable | Abbrev. | Range | Mean ± SD | F | p-value |
| Total Nitrogen (mg/L) | TN | 0.47~2.71 | 1.23±0.55 | 20.80 | <0.001 |
| Total Phosphorus (mg/L) | TP | 0.01~0.3 | 0.08±0.06 | 6.65 | 0.003 |
| Phosphate (mg/L) | PO4 | 0.01~0.06 | 0.01±0.01 | 22.20 | <0.001 |
| Ammonia Nitrogen (mg/L) | NH3-N | 0.07~1.56 | 0.4±0.32 | 6.68 | 0.003 |
| Chemical Oxygen Demand (mg/L) | COD | 0.7~7.52 | 4.06±1.93 | 52.19 | <0.001 |
| Chlorophyll-a (mg/L) | Chl-a | 0.41~38.75 | 8.07±7.43 | 5.04 | 0.010 |
| Water Temperature (°C) | T | 5.82~34.09 | 20.92±10.75 | 1164.83 | <0.001 |
| Dissolved Oxygen (mg/L) | DO | 2.1~12.05 | 6.05±3.49 | 426.54 | <0.001 |
| Hydrogen Ions | pH | 5.38~200 | 36.4±60.06 | 1.72 | 0.190 |
| Conductivity (μs/cm) | CON | 0.07~172 | 73.01±54.13 | 7.87 | 0.001 |
| Water Depth (m) | WD | 6.78~18.23 | 12.84±5.03 | 202632.17 | <0.001 |

**Table S7** One-way ANOVA summary of environmental variables at different times in Changhuchi. P-values: the significance of differences in environmental factors across time.

| Changhuchi | | | | | |
| --- | --- | --- | --- | --- | --- |
| Environment variable | Abbrev. | Range | Mean ± SD | F | p-value |
| Total Nitrogen (mg/L) | TN | 2.11±1.39 | 0.62~4.71 | 52.25 | <0.001 |
| Total Phosphorus (mg/L) | TP | 0.03±0.02 | 0.01~0.06 | 15.81 | <0.001 |
| Phosphate (mg/L) | PO4 | 0.01±0.01 | 0.01~0.04 | 2.22 | 0.139 |
| Ammonia Nitrogen (mg/L) | NH3-N | 0.89±1.12 | 0.07~3.58 | 45.79 | <0.001 |
| Chemical Oxygen Demand (mg/L) | COD | 3.67±1.09 | 1.98~5.68 | 36.57 | <0.001 |
| Chlorophyll-a (mg/L) | Chl-a | 3.28±3.69 | 0.18~11.19 | 4.39 | 0.026 |
| Water Temperature (°C) | T | 20.01±8.63 | 6.25~31.26 | 1038.86 | <0.001 |
| Dissolved Oxygen (mg/L) | DO | 8.54±3.21 | 2.61~12.05 | 171.37 | <0.001 |
| Hydrogen Ions | pH | 6.43±0.29 | 5.79~7.00 | 6.44 | 0.008 |
| Conductivity (μs/cm) | CON | 178.87±100.76 | 35.9~294.1 | 1359.37 | <0.001 |
